# Antimicrobial peptide DP7 alleviates dextran sulfate sodium (DSS)‐induced colitis via modifying gut microbiota and regulating intestinal barrier function

**DOI:** 10.1002/mco2.70085

**Published:** 2025-01-30

**Authors:** Binyan Zhao, Hongyou Zhou, Ke Lin, Jie Xu, Bailing Zhou, Daoyuan Xie, Jing Ma, Lei Yang, Chunyan Su, Li Yang

**Affiliations:** ^1^ Department of Biotherapy, Cancer Center and State Key Laboratory of Biotherapy West China Hospital, Sichuan University Chengdu China; ^2^ Sichuan Institute for Drug Control The People's Republic of China Chengdu China

**Keywords:** antimicrobial peptide, DP7, Inflammatory bowel disease, intestinal microbiota

## Abstract

Inflammatory bowel diseases (IBDs), such as Crohn's disease (CD) and ulcerative colitis (UC), represent a growing global health concern. Restoring the balance of the gut microbiota, a crucial factor in intestinal health, offers potential for treating IBD. DP7, a novel antimicrobial peptide with potent antibacterial activity, was investigated for its anti‐inflammatory effects in a dextran sulfate sodium (DSS)‐induced UC mouse model. DP7 significantly ameliorated key disease parameters, including disease activity index, weight loss, and shortened colon length, while preserving colonic epithelial integrity and reducing inflammatory infiltration. Further analysis revealed potential targets of DP7, highlighting the significant role of *Muribaculaceae* bacteria during inflammatory states. To further explore the role of the gut microbiota in DP7's efficacy, fecal microbiota transplantation (FMT) was performed using feces from DP7‐treated mice. FMT successfully ameliorated colitis in recipient mice, providing further evidence for the crucial role of the gut microbiome in IBD treatment and DP7's ability to modulate the gut microbiota for therapeutic benefit. Moreover, our findings suggest that DP7's modulation of the immune system is intricately linked to the complex microbial environment. Our findings demonstrate that DP7 effectively mitigates inflammation, attenuates barrier dysfunction, and shapes the gut microbiota, suggesting its potential as a therapeutic agent for UC.

## Graphical abstract

DP7 effectively alleviated dextran sulfate sodium (DSS)‐induced colitis in mice. The potential mechanisms include enhancing gut barrier integrity, reducing proinflammatory cytokine secretion, and modulating gut microbiota composition. These findings highlight DP7's therapeutic potential for treating inflammatory bowel diseases by targeting both immune response and microbiota homeostasis.

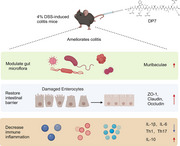

## 1 INTRODUCTION

Inflammatory bowel disease (IBD) represents a group of chronic and relapsing disorders characterized by inflammation of the intestinal mucosa, and it has devolved into a global disease with increasing prevalence on every continent.^1^ While the exact etiology of IBD remains elusive, dysregulated immune responses and alterations in the gut microbiota are considered pivotal factors driving disease pathogenesis.^2^, ^3^

Several observations in human subjects provide evidence for the involvement of the microbiome in individuals with IBD. Notably, antibiotic therapy has been shown to be effective in treating specific IBD phenotypes.^4^, ^5^ However, the utilization of antibiotics in clinical practice is constrained by diverse trials that frequently yield contradictory outcomes.^6^ Furthermore, there is concern about the potential development of antibiotic resistance, which has been underscored by past studies.^7^, ^8^ Consequently, the pursuit of innovative, effective, and secure treatment approaches stands as a pivotal area of research within the realm of IBD.

Antimicrobial peptides (AMPs) exhibit strong antimicrobial activity and a low rate of resistance accumulation, making them promising alternatives to antibiotics. In recent years, a growing body of evidence has revealed the therapeutic potential of AMPs in the context of IBD, particularly in dextran sulfate sodium (DSS)‐induced colitis models.^9^ As the field has advanced, researchers have increasingly dedicated their efforts to unraveling the intricate interplay between the gut microbiota, host immunity, and AMPs in the context of colonic inflammation. This endeavor has the potential to unveil novel targets for therapeutic interventions and provide insights into the broader implications of AMP‐based strategies in managing IBD. High‐impact studies by Sun et al.^10^ underscore the integral role of AMPs in shaping the gut microbe composition and thus maintaining gut health and highlights the potential of these molecules as therapeutic candidates. Moreover, recent investigations by Jia et al.^11^ have delved into the molecular mechanisms underlying the modulation of immune responses by gut microbes, revealing intricate crosstalk that may hold the key to ameliorating colitis progression. In conclusion, an expanding body of research has examined the therapeutic potential of AMPs in DSS‐induced colitis. By elucidating the multifaceted interactions between AMPs, the gut microbiota, and immune responses, this study aims to contribute to the development of innovative strategies for the management of IBD.

Our team previously engineered and refined a groundbreaking AMP named DP7 (VQWRIRVAVIRK‐NH2).^12^, ^13^, ^14^, ^15^ Compared with its concise sequence of 12 amino acids, DP7 has wide‐ranging antibacterial properties, and its development has been guided by an innovative amino acid‐based predictive methodology. Our past investigations vividly revealed the effectiveness of DP7 in curbing the proliferation of the gram‐positive bacterium *Staphylococcus aureus* while maintaining a commendable level of cytocompatibility with human cells. Furthermore, DP7 exhibited a synergistic effect when it was combined with vancomycin and azithromycin to combat multidrug‐resistant (MDR) bacteria.^12^, ^14^, ^16^ The administration of DP7 significantly alleviated DSS‐induced colitis in mice and facilitated the restoration of intestinal barrier function. These findings offer empirical evidence for the therapeutic effect of DP7 against DSS‐induced colitis. Additionally, 16S rRNA sequencing indicated that these effects may be linked to the remodeling of the gut microbiota by DP7. Moreover, DP7 reduced the abundance of *Alloprevotella* bacteria and increased the abundance of *Muribaculaceae* in the mice. In conclusion, DP7 has promising potential as a prospective therapeutic agent for treating intestinal inflammatory disorders.

## 2 RESULTS

### 2.1 Effects of antibiotics on colon morphology and inflammatory status

The mice were administered various concentrations of DSS in their drinking water for 7 days, followed by a 3‐day observation period with normal drinking water to establish a colitis mouse model. DSS treatment resulted in significant weight loss, an increase in the disease activity index (DAI) score, a reduction in colon length, and tissue morphological changes (Figure S1A–D). On the basis of these data, we conclude that the 4% DSS concentration is the optimal dosage for inducing the colitis model.

**FIGURE 1:**
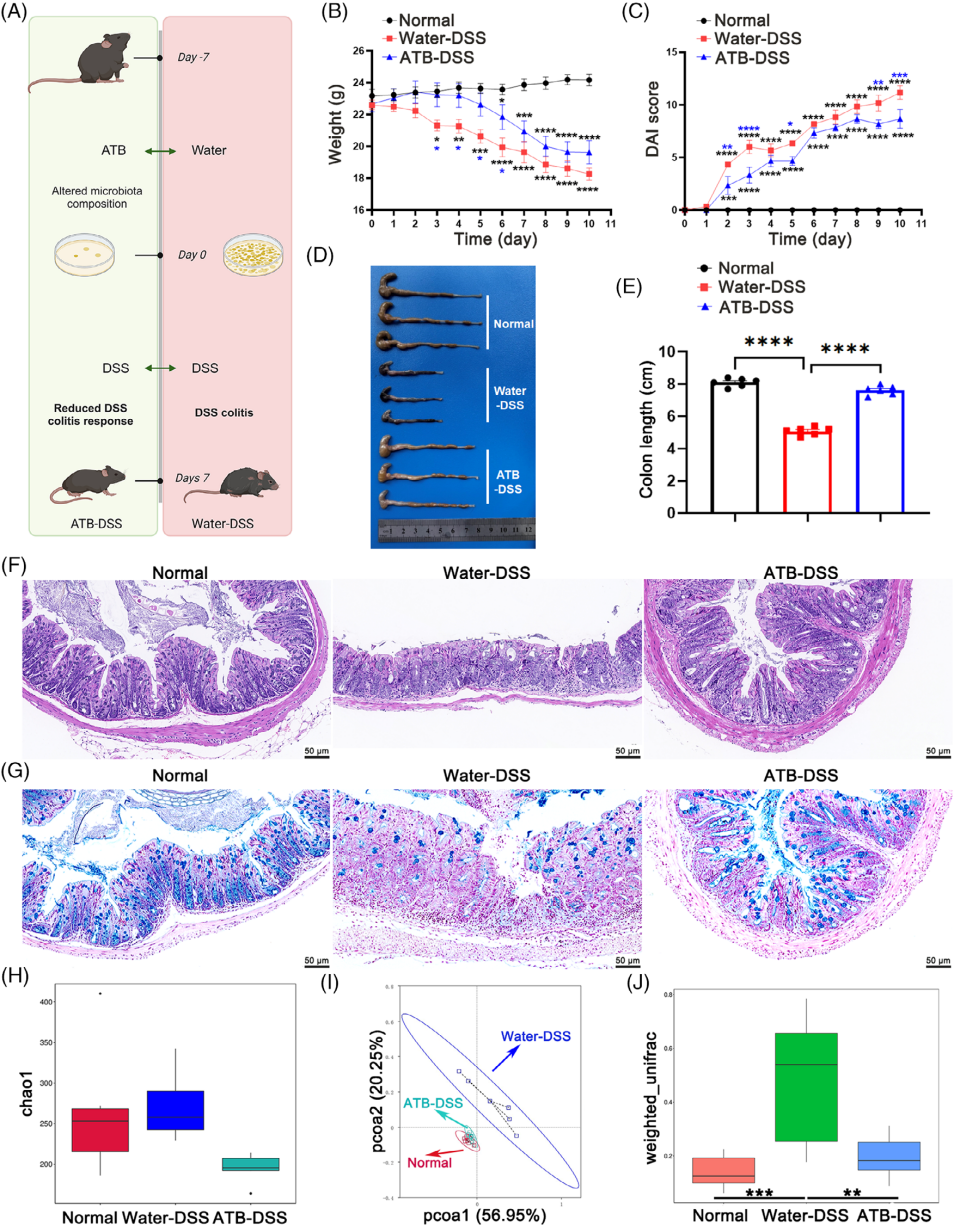
Effects of antibiotics pretreatment on colon morphology and inflammatory status. (A) Schematic of the experimental design employed in this investigation. (B) Weight monitoring results during the trial. (C) Kinetics of disease activity index (DAI) scores throughout the study. (D) Macroscopic images and (E) lengths of colons in each group. (F) Representative images of hematoxylin and eosin (H&E)‐stained colons. The dextran sulfate sodium (DSS) group exhibited severe mucosal destruction, along with submucosal edema and extensive inflammatory cell infiltration. (G) Representative images of the inner mucus layer of Alcian blue (AB)‐stained colon sections. AB staining revealed the secretory function of the intestinal cells. Following DSS treatment, intestinal secretory function was markedly impaired, whereas ATB‐treated mice exhibited less damage. (H) The Chao1 index for different groups. (I) The principal coordinate analysis (PCoA) plot was analyzed with permutational multivariate analysis of variance (PERMANOVA) for all three groups. (J) Box plots of beta diversity from the intergroup difference analysis provide visual information on the median, dispersion, maximum, minimum, and outliers of sample similarity within groups. Tukey's test was then used to assess the significance of differences in the beta diversity of species between groups. Scale bars are 50 µm (*n* =  6 per group).

On the basis of clinical observations, in animal experiments in which antibiotics were pretreated (Figure 1A), the DSS group exhibited notable characteristics (Figure 1B–E), and a reduction in food intake (Figure S4C). These manifestations collectively underscore the successful establishment of the experimental model. Notably, pretreatment with antibiotic supplement (ATB–DSS) mitigated these symptoms. Histopathological evaluation (20×) of distal colonic tissue via hematoxylin and eosin (H&E) staining derived from the DSS group revealed pronounced edema, extensive mucosal impairment, and notable infiltration of inflammatory cells. In contrast, the ATB–DSS group exhibited diminished infiltration of inflammatory cells within the mucosal layer, coupled with clear tissue architecture and an abundance of glands (Figure 1F). Histological evaluation of colon pathology was conducted by examining the number of ulcers, epithelial cell changes, and the degree of inflammatory infiltration in colon tissue (Figure S2A). The findings indicated that in the ATB–DSS group, there was a significant reduction in these pathological markers. The results demonstrated that pretreatment with antibiotics led to less severe morphological alterations in the intestinal tissue of mice compared to the DSS group. To assess the effects of both DSS and ATB on the colonic mucosal barrier, alcian blue (AB) staining was employed to quantify goblet cells that secreted mucins within the colonic epithelium (Figure 1G). Antibiotic treatment alleviated the reduction in goblet cells caused by DSS (Figure S2B).

The intestinal epithelium is crucial for mucosal barrier integrity. To evaluate the impact of antibiotics on this barrier, we conducted in vivo experiments. Assessing intestinal tight junctions (TJs) and related protein expression is a standard method for evaluating epithelial barrier damage.^17^ Western blotting (Figure S2C) and reverse transcription‐polymerase chain reaction (RT‐PCR; Figure S2D) revealed that ZO‐1, claudin, and occludin were expressed at lower levels than normal in the DSS group. Furthermore, immunofluorescence (IF) was performed on these tissues, which revealed that DSS reduced the levels of ZO‐1, claudin, and occludin in the colon (Figure S2E). However, antibiotic supplementation resulted in increased colonic TJ protein levels in the context of colitis. Therefore, antibiotics alleviate colitis by upregulating TJ proteins and preserving intestinal barrier function.

Antigen‐specific T cells, particularly T helper 1 (Th1) and T helper 17 (Th17) cells, play a vital role in maintaining mucosal immunity. However, they are implicated in the pathogenesis of colitis.^18^, ^19^ Consequently, we examined changes in immune cells within the mesenteric lymph nodes (MLNs) and detected a significant increase in the proportions of Th1 (Figure S3A) and Th17 (Figure S3B) cells in DSS‐induced colitis mice, which was notably controlled in the antibiotic‐treated group. Previous studies have suggested that the imbalance between the gut microbiota and immune responses plays a crucial role in IBD. Typically, regulatory T cells (Tregs) are essential for maintaining intestinal immune homeostasis, and mice deficient in Tregs are more susceptible to intestinal inflammation. DSS treatment resulted in a reduction in Treg cell numbers, whereas mice pretreated with antibiotics presented Treg cells that were nearly unaffected by DSS (Figure S3C). These findings suggest that antibiotic treatment can restore the balance of Th17/Treg cells in adaptive immunity to some extent.

Furthermore, the expression of the cytokines in colonic tissue was assessed via RT‐PCR (Figure S3D–F) and ELISA (Figure S3G–I). Compared with those in the other groups, the levels of the proinflammatory cytokines IL‐6 and IL‐1β were greater in the DSS group, whereas the levels of the anti‐inflammatory cytokine IL‐10 were lower. Previous studies have demonstrated that the expression of CCL9, CXCL1, and CXCL2 is significantly increased in the colonic epithelial cells of experimental colitis mice and ulcerative colitis (UC) patients. These chemokines play critical roles in the recruitment of monocytes and granulocytes, thereby maintaining inflammation in UC. The levels of these genes correlate with disease activity in UC patients and may serve as markers of therapeutic response. Therefore, we utilized RT‐PCR to assess the expression levels of these chemokines in colonic tissue. Notably, the water–DSS‐treated group exhibited significant upregulation of these chemokines, whereas this increase was controlled in the ATB–DSS‐treated group (Figure S3J–L). During UC, neutrophils are typically activated at the inflamed intestinal site.^20^, ^21^ Therefore, we employed IF to detect apoptotic cells (TUNEL) and the neutrophil‐specific marker myeloperoxidase (MPO) in the colonic tissue of the different groups of mice. The results showed that ATB–DSS treatment reduced the proportion of apoptotic cells and neutrophil infiltration in intestinal tissue and that changes in chemokine levels may underlie the controlled neutrophil infiltration (Figure S3M).

To explore the microbiota's role in colitis, we used antibiotics to eliminate intestinal microbes and confirmed successful clearance (Figure S4A,B). Our findings also demonstrate that the gut microbiota is indeed associated with the development and remission of colitis. Moreover, we investigated the influence of antibiotics on the composition of the intestinal microbiota in DSS‐treated mice. Compared with the remaining two groups, the water–DSS group presented increased α diversity, potentially indicative of bacterial infection. Conversely, the antibiotic‐treated group presented a reduction in α diversity, although the difference was not statistically significant (Figure 1H). Principal coordinate analysis (PCoA) revealed distinct microbial configurations between the ATB–DSS and water–DSS groups, whereas discernible separation was not observed between the normal and ATB–DSS groups (Figure 1I). Beta diversity analysis, which was predicated on weighted UniFrac distances, corroborated analogous patterns between the two sample groups (Figure 1J). This observation indicates that antibiotic pretreatment and DSS treatment indeed disrupted the gut microbiota, as there was a distinct difference in the gut microbiota composition between the water–DSS group and the ATB–DSS group. The composition of gut microbiota significantly impacts the effect of DSS‐induced colitis, as evidenced by the milder inflammation symptoms observed in germ‐free mice.

The similarity in gut microbiota composition between the ATB‐treated group and the normal group in the PCoA plot, with no significant distinction, may also be attributed to the changes induced by DSS. DSS‐induced colitis disrupts gut homeostasis, including microbial and immune imbalances, leading to a substantial deviation in gut microbiota between the DSS group and the normal group. In contrast, the PCoA analysis shows that the ATB‐treated group is more similar to the normal group, possibly because the absence of gut microbiota makes it more challenging for DSS to cause gut homeostasis imbalance, thereby delaying the onset and severity of colitis.

### 2.2 DP7 significantly alleviated the severity of colon disease in mice with colitis

**FIGURE 2:**
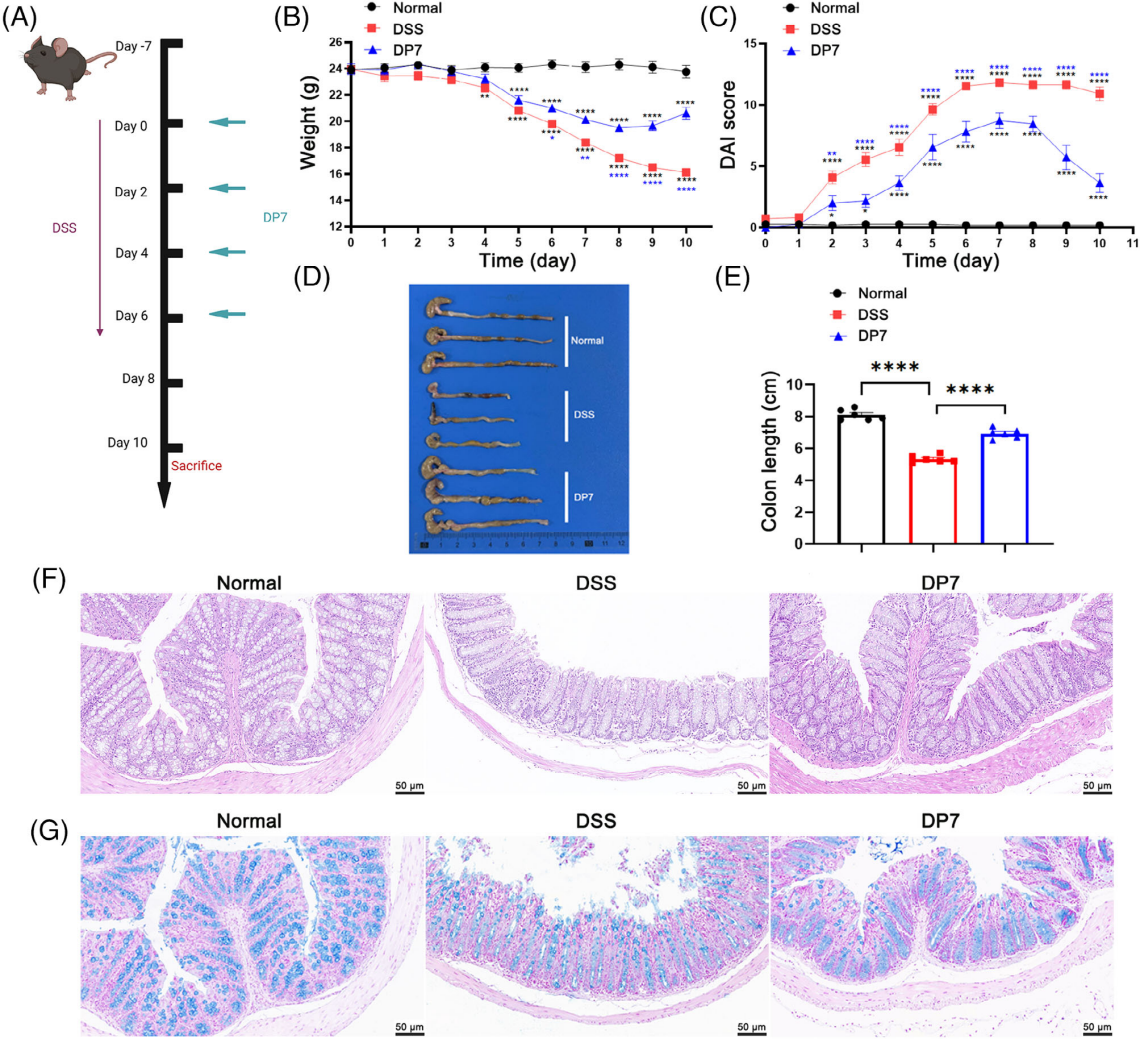
DP7 significantly alleviated the severity of colon disease in the mice with colitis. (A) Schematic of the experimental design used in this investigation. (B) Changes in body weight of the mice. (C) Disease activity index (DAI) score changes over time. (D) Representative photographs of mouse colons at the end of the experiment. (E) Lengths of the colons from each group. (F) Representative colon images by hematoxylin and eosin (H&E) staining demonstrated that colonic inflammation and injury were clearly evident in the dextran sulfate sodium (DSS)‐treated group, whereas H&E staining of the DP7 group revealed a near‐normal appearance. (G) Representative images of the inner mucus layer of Alcian blue (AB)‐stained colon sections. AB staining indicated that the secretory function of the intestinal cells in the DP7 group had recovered well. Scale bars are 50 µm (*n* =  6 per group).

To investigate the mitigating effect of DP7 on DSS‐induced colitis, mice were treated with a 4% aqueous solution of DSS for 7 days to induce experimental colitis, followed by intravenous administration of DP7 at the optimal dose previously reported.^12^, ^14^, ^16^ During model establishment, the mice experienced weight loss and worsening diarrhea, with blood and mucus in their stools. Compared to the DSS‐only group, DP7 treatment significantly reduced weight loss, increased DAI score (Figure 2B,C) and increased food intake (Figure S8A). DP7 also reversed colon shortening caused by DSS (Figure 2D,E). The DSS group exhibited disrupted glandular architecture, fewer goblet cells, and extensive inflammatory cell infiltration (H&E and AB staining, Figure 2F,G), but DP7 treatment mitigated these pathological changes and significantly improved histopathological scores (Figure S6A,B).

In conclusion, these results suggest that DP7 may ameliorate colitis symptoms and colon damage.

### 2.3 DP7 restored the intestinal tight junction structure and modulated serum cytokine and colon cytokine gene expression

Additionally, we evaluated the influence of DP7 on the colonic mucosal barrier via detection of colonic TJ protein expression levels via western blotting (Figure S6C), RT‐PCR (Figure S6D), and IF (Figure S6E). ZO‐1, claudin‐1, and occludin were notably lower in the DSS group than in the other groups, and the colonic TJ protein levels of the colitis model mice were increased following the administration of DP7.

Previous studies have shown that both Th1 and Th17 cells are involved in intestinal inflammatory processes, mainly through their production of proinflammatory cytokines. We investigated alterations in immune cells within the MLNs and observed a significant decrease in Th1 and Th17 cells in the DP7 group (Figure S7A,B). Concurrently, the proportion of regulatory T (Treg) cells involved in the suppression of inflammation increased in mice with colitis following DP7 treatment (Figure S7C). DP7‐treated mice had significantly reduced plasma levels of IL‐1β and IL‐6, with a concurrent increase in IL‐10 (Figure S7D–I). Similarly, injection of DP7 significantly suppressed the increase in the levels of the chemokines CCL9, CXCL1, and CXCL2 in the colons of DSS‐induced mice (Figure S7J–L). Consistent with these findings, the concentrations of apoptotic cells and MPO in the murine colon significantly increased in response to DSS exposure; however, the introduction of DP7 effectively reduced this increase (Figure S7M).

These findings show that intravenous DP7 administration mitigated DSS‐induced colonic inflammation and impaired mucosal barrier function.

### 2.4 Microbiota diversity analysis

**FIGURE 3:**
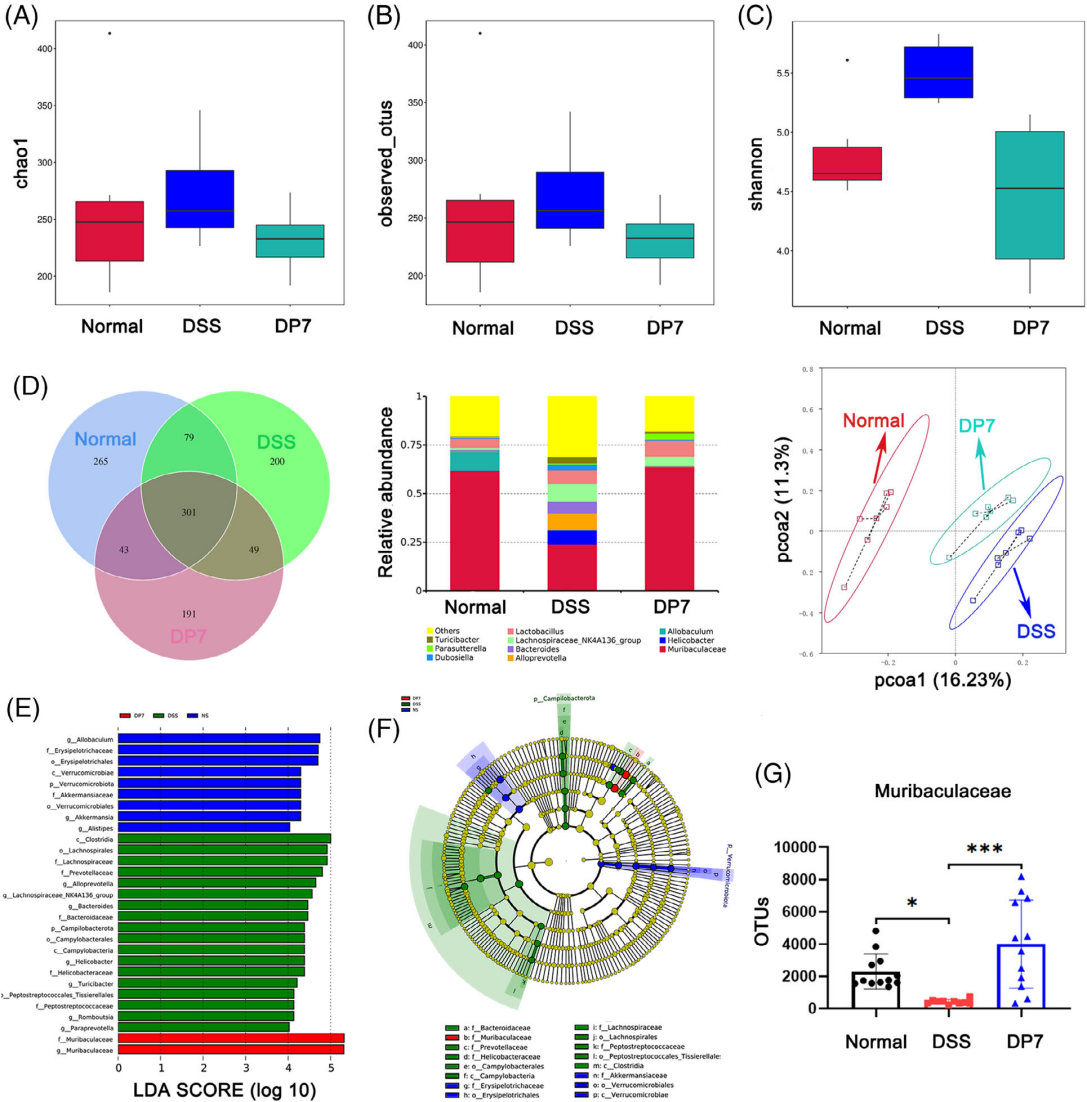
Changes in the diversity and structure of the microbiota. (A) Alpha diversity comparisons of the microbial communities. *p <  0.05; ***p <  0.001 compared with the normal group (Wilcoxon rank‐sum test). (B) Venn diagrams were constructed to analyze the shared and specific amplicon sequence variants (ASVs) between various groups. (C) The relative abundance of bacterial genera found in feces. (D) Principal coordinate analysis (PCoA) plots were used to assess the impact of DP7 on the intestinal flora structure of the mice with colitis, with statistical significance tested via permutational multivariate analysis of variance (PERMANOVA; *n* =  6 per group). Analysis of discrepancies in the microbial taxa displayed by LEfSe: (E) linear discriminant analysis (LDA) histogram and (F) evolutionary branching maps (phylogenetic distribution). (G) Examples of bacteria for which the abundance increased following DP7 treatment. DSS, dextran sulfate sodium. OTU, operational taxonomic unit.

We investigated the impact of DP7 treatment on the gut microbiota through 16S rRNA gene sequencing. Analysis of community richness, or α diversity, via the Chao 1, observed_otus, and Shannon indices revealed increased diversity in the DSS group, whereas the diversity in the DP7 group was decreased, with no significant differences between the groups (Figure 3A). Similarly, the Venn diagram (Figure 3B) also illustrates comparable outcomes. Notably, DSS substantially increased the relative abundance of pathogenic bacteria at the genus level (Figure 3C). PCoA based on the Bray–Curtis distance revealed a distinct difference in the gut microbiota structure between the normal and DSS groups and between the DP7 and DSS groups, suggesting that DP7 has a significant effect on the gut microbiota structure in colitis mice (Figure 3D). LEfSe analysis revealed that the fecal bacterial taxa significantly enriched by the different treatments. DP7 injection significantly enriched the bacterial genus *Muribaculaceae*, whereas six taxa—*Alloprevotella*, *Lachnospiraceae_NK4A136_group*, *Bacteroides*, *Helicobacter*, *Turicibacter*, and *Romboutsia*—were enriched only in the DSS group (Figure 3E). The corresponding evolutionary branching diagrams are shown in Figure 3F. In addition, specific quantitative changes were observed (Figure 3G).

DP7 from intravenous injection affected the intestinal flora of the mice with colitis. These findings indicate that changes in the intestinal flora caused by DP7 may play a significant role in reducing DSS‐induced colitis.

### 2.5 Correlation analysis between specific microbiota and disease indicators

**FIGURE 4:**
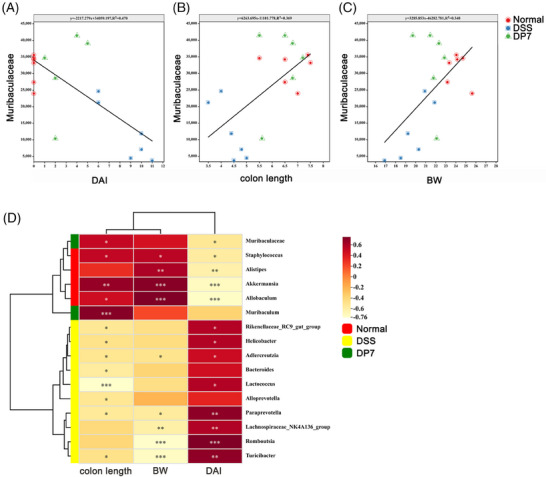
Correlation analysis between the microbiota and inflammation‐associated parameters. (A–C) Linear regression relationships between the amplicon sequence variants (ASVs) of *Muribaculae* and disease activity index (DAI), colon length, and body weight (*n* =  5 per group). (D) Correlation matrix between specific microbiota genera and inflammation‐associated parameters (Spearman correlation). DSS, dextran sulfate sodium. BW, body weight.

To further identify the relationship between the microbiota and inflammation, correlation analysis was used. Linear regression analysis was conducted on a significantly enriched bacterial genus from the DSS group and DP7 group. The results revealed that the amplicon sequence variants (ASVs) of *Muribaculae*, which were considerably enriched in the DP7 group, were positively correlated with colon length and weight and negatively correlated with DAI (Figure 4A–C), which was coincident with the results of linear regression analysis (Figure 4D). These results suggest that the administration of DP7 affects the proportion of intestinal flora and that significantly increased intestinal flora is associated with symptomatic relief. These findings suggest that we should investigate the relationship between changes in the gut flora induced by DP7 and the efficacy of treatment.

### 2.6 Compared with DSS–FMT, DP7–FMT contributed more strongly to alleviating colitis

**FIGURE 5:**
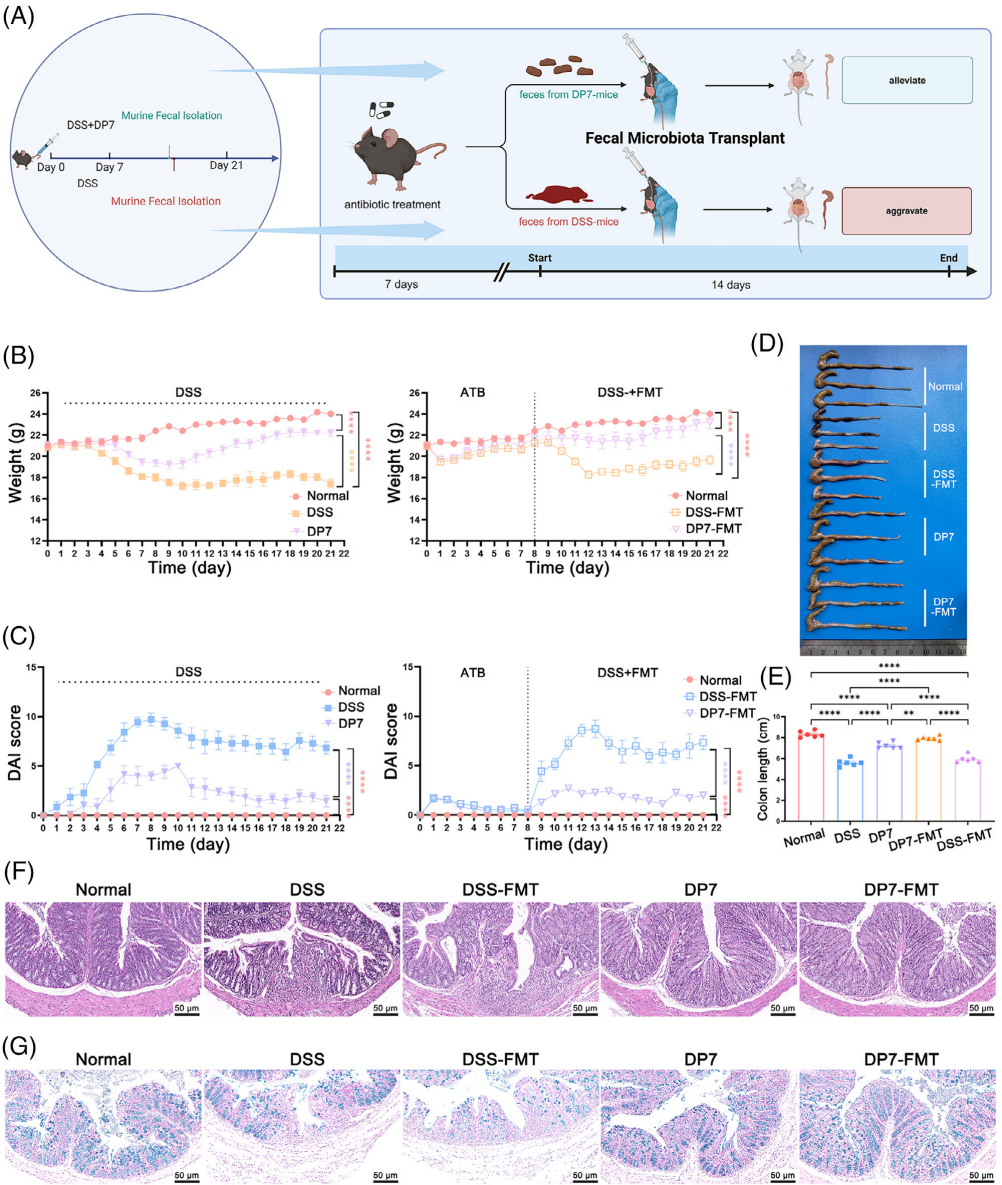
Compared with dextran sulfate sodium (DSS)‐derived fecal microbiota, microbiota transplantation from mice treated with DP7 relieves colitis better. (A) Schematic diagram illustrating the experimental setup and preparation employed in this investigation. (B) Daily body weight changes following treatment. The weights of the mice in the DP7‐FMT group were significantly greater than those in the DSS–FMT group. (C) Disease activity index (DAI) scores following treatment. The DAI scores in the DP7–FMT group were significantly lower than those in the DSS–FMT group. (D) Macroscopic images of the colon. The mice in the DP7–FMT group presented the least severe colon damage and inflammation. (E) Colon length. The colons of the mice in the DP7–FMT group were significantly longer than those in the DSS and DSS–FMT groups. (F) Hematoxylin and eosin (H&E)‐stained colon sections. Colon injury was the mildest in the mice in the DP7‐FMT group among the five groups. (G) Alcian blue (AB)‐stained colon sections. Scale bars are 50 µm (*n* =  6 per group).

Given the potential interaction between the gut microbiota and DP7, it was hypothesized that DP7 alleviates colitis mainly by regulating the gut microbiota, thereby reducing host inflammation and promoting intestinal barrier repair. The effect of the DP7‐mediated microbiota on colitis in mice was then validated in this animal study, which was achieved by transplanting fecal microbiomes from mice into mice that had been treated with DSS (Figure 5A). The efficacy of DP7 in inducing remission in a DSS‐induced colitis model was initially evaluated. We subsequently conducted a comparative analysis of control FMT and untreated (DSS–FMT) control mice, with the objective of further substantiating the reduction in the specific gut microbiome of DP7‐treated donors.

Notably, compared with DSS and DSS–FMT, DP7 and DP7–FMT more profoundly alleviated the symptoms of acute colitis, as indicated by a decrease in body weight loss, DAI score, and histological score and an increase in colonic length (Figure 5B–E). In addition, the results of H&E staining and AB staining also confirmed that DP7–FMT treatment effectively reduced the intestinal tissue damage induced by DSS. Intestinal morphological studies revealed that colonic crypt height and myometrial width were significantly greater in the DP7–FMT mice than in the control mice (Figure 5F). AB staining revealed a significant increase in the number of mucus‐producing goblet cells in the colon of DP7–FMT mice compared with that of the control mice (Figure 5G).

The colitis‐induced Th1 and Th17 cells and associated cytokines are responsible for the severity of the development of subsequent colitis lesions. Compared with the DSS group, the DSS–FMT group presented significant increases in Th1 and Th17 cells. The DP7–FMT group was similar to the DP7 group, controlling the increase in Th1 and Th17 cells while restoring the normal expression level of Tre in vivo (Figure S9A–C). In addition, the IL‐6 and IL‐1β levels were increased, and the IL‐10 levels were decreased in the colon tissue and plasma of the mice in the DSS group and DSS–FMT group (Figure S9D–I), as were the CCL9, CXCL1, and CXCL2 (Figure S9J–L) levels. Notably, DP7 and DP7–FMT reduced the expression levels of related proinflammatory cytokines and chemokines and reduced apoptosis and neutrophil infiltration in colon tissue (Figure S9M).

### 2.7 DP7–FMT was more effective than DSS–FMT in alleviating colonic barrier damage

The intestinal epithelium separates the contents of the intestinal cavity from those of the mucosal immune system, thereby acting as an important defense line to protect the homeostasis of the intestinal microbiome and reduce the intestinal inflammatory response. Compared with DSS–FMT treatment, DP7–FMT treatment resulted in superior mucus distribution and maintained the integrity of the close colonic connections (Figure 5F,G). Furthermore, histological scoring (Figure S10A) and goblet cell counts (Figure S10B) also indicated that DP7–FMT treatment, similar to DP7 alone, alleviated intestinal tissue damage. The expression level of ZO‐1, occludin, and claudin were quantified via western blot (Figure S10C), RT‐PCR (Figure S10D), and IF (Figure S10E) techniques. These results demonstrated that DP7–FMT treatment increased the expression of TJ proteins. In conjunction with the results of the morphological analysis, these findings suggest that DP7–FMT may enhance the protective effect. The alleviation of colitis by means of enhanced intestinal barrier function.

### 2.8 Analysis of the intestinal flora structure after FMT

**FIGURE 6:**
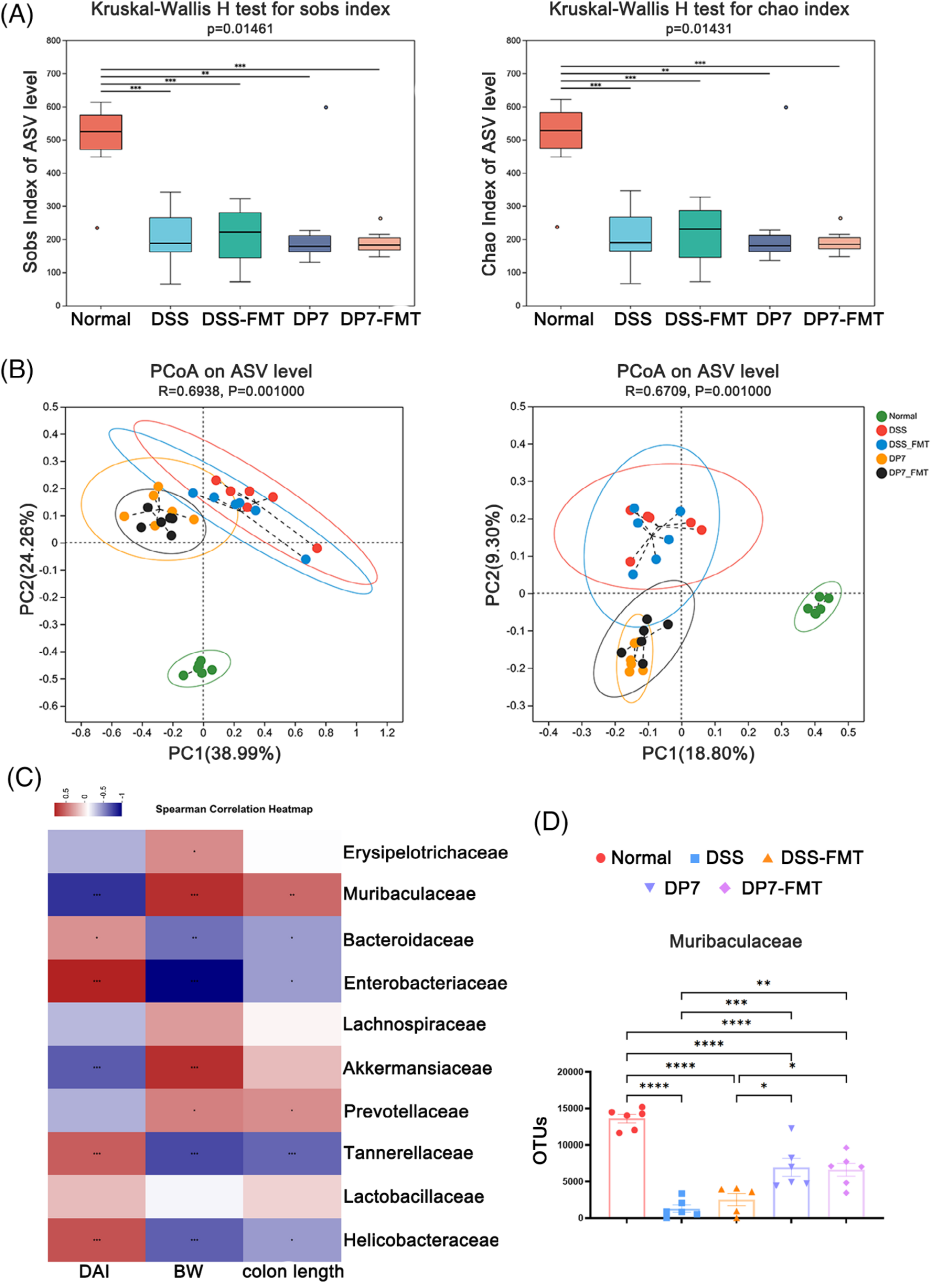
DP7–FMT reverses microbiota dysbiosis. (A) Alpha diversity of the gut microbiota in different groups, analyzed using the Sobs index and Chao index. (B) Beta diversity of the gut microbiota in different groups, visualized using principal coordinate analysis (PCoA). (C) Correlation analysis between the abundance of enriched bacteria in the DP7 group and body weight, colon length, and disease activity index (DAI) score. Red indicates positive correlations, and blue indicates negative correlations. (D) Differential abundance of *Muribaculaceae* across all groups, based on the relative abundance of each bacterial species (*n*  =  6 per group). ASV, amplicon sequence variant; DSS, dextran sulfate sodium.

Compared with those of donors, the Sobs and Chao1 indices of the ASV level were not significantly impacted by FMT in mice with colitis compared with donor (Figure 6A). PCoA revealed the change in structure among all the five groups (Figure 6B): the structure of the gut microbiota in the recipient mice exhibited notable similarities to that of the donor microbiota. These findings indicate that the fecal bacteria transplantation technique can transfer the intestinal flora of mice treated with DP7 to new mice and that the effects in mice receiving fecal bacteria transplantation are similar to those in those receiving DP7 (Figure 5). Moreover, correlation analysis indicated that *Muribaculaceae* was positively correlated with relieved colitis symptoms (Figure 6C). Overall, DP7–FMT alleviated colitis and colonic barrier damage, enriched *Muribaculaceae* were also induced by DP7–FMT (Figure 6D).

### 2.9 DP7 regulates immune homeostasis in colonic tissue in a complex intestinal microbiome environment

**FIGURE 7:**
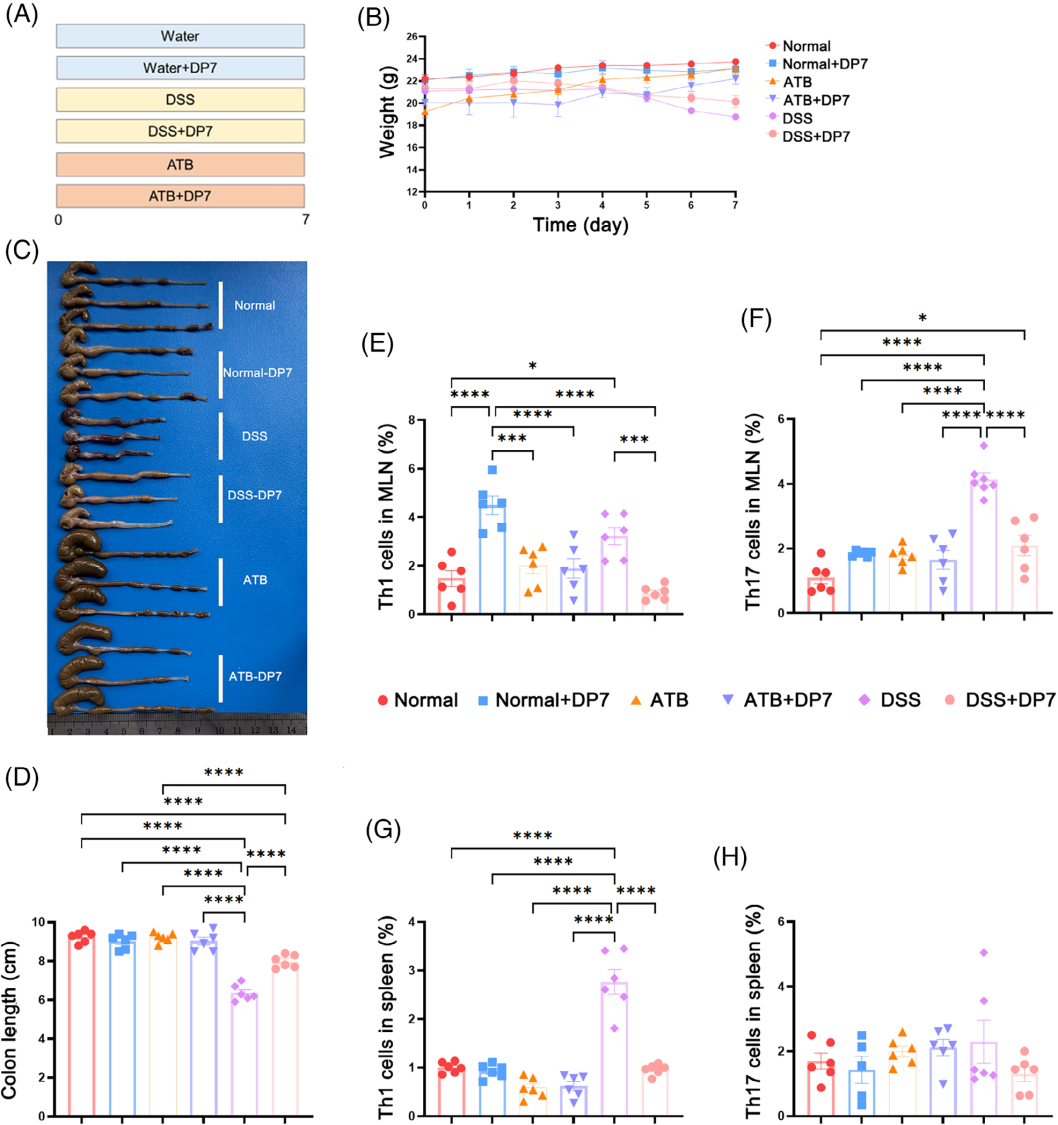
The effects of DP7 on immune responses are closely associated with changes in gut microbiota. (A) Experimental setup diagram. (B) Daily body weight changes following treatment. (C) Macroscopic images and (D) lengths of colons in each group. Flow cytometry was used to identify changes in the proportions of Th1 and Th17 cells in mesenteric lymph nodes (E, F) and the spleen (G, H) (*n* = 6 per group). DSS, dextran sulfate sodium; MLN, mesenteric lymph node.

To investigate the impact of DP7 on the immune system of mice, a study was conducted (Figure 7A). The body weights of the mice and macroscopic images of the colons were recorded (Figure 7B,C). Symptoms of DSS‐induced colonic shortening were observed, whereas DP7 treatment effectively alleviate, these symptoms, which is in line with previous experimental results. The administration of antibiotics resulted in an enlargement of the cecum (Figure 7D). The results demonstrated that DP7 had disparate effects on immune cells in normal mice, mice with DSS‐induced colitis, and pseudo‐germ‐free mice. The gut microbiota of these mice presented distinct signatures. In normal mice, DP7 treatment resulted in an increase in the proportion of Th1 cells in the MLNs. However, there was no significant effect on Th17 cells. The administration of DP7 did not affect the proportion of Th1 and/or Th17 cells in the MLN of antibiotic‐treated mice (pseudo‐germ‐free mice; Figure 7E,F). In mice with DSS‐induced colitis, as observed in previous experimental results, DSS resulted in a significant increase in Th1 and Th17 cells and a decrease in Treg cells. The administration of DSS disrupted immune homeostasis within the intestinal tract, whereas DP7 treatment was associated with a reduction in Th1 and Th17 cells and an increase in Treg cells. These findings indicate that DP7 treatment facilitates the restoration of intestinal immune homeostasis. These results demonstrate the effect of DP7 on the intestinal immune system of mice, provided that there are microorganisms present in the gut, as DP7 is immune to the gut of pseudo‐germ‐free mice.

The successful establishment of the pseudo‐germ‐free mouse model was validated through fecal culture (Figure S12A) and quantitative fecal DNA analysis (Figure S12B), confirming the complete clearance of bacteria from the mice following antibiotic treatment. Additionally, we observed the alterations in spleen immune cells. Unexpectedly, there was no significant difference in the number of Th1, Th17, or Treg cells in the spleen between normal mice with intestinal microorganisms and pseudo‐germ‐free mice after DP7 injection. However, there was a significant increase in Th1 cells due to DSS, which then recovered somewhat after DP7 treatment. With respect to Treg cells, DP7 treatment had no significant effect on either systemic or intestinal immunity (Figure S12C,D).

These findings indicate that DP7 has a more pronounced effect on the intestinal immune system than on systemic immunity, thereby facilitating the restoration of immune homeostasis imbalance induced by DSS, which is contingent upon the presence of a normal gut microbiota. Notably, the immune system of DP7‐treated pseudo‐germ‐free mice remained unaltered.

## 3 DISCUSSION

Gut bacteria are crucial for normal DSS‐induced colitis development; their absence significantly decreases DSS colonic inflammation and impairs barrier function.^22^ Although limited, some clinical data support the efficacy of microbiota‐targeted therapeutic strategies for IBD patients.^23^, ^24^ This includes the use of antibiotics to prevent postoperative recurrence in Crohn disease (CD) patients and to treat pouchitis and perianal disease.^5^ Antibiotics reduce the number of luminal microorganisms and alter microbial composition.^25^ Rifaximin is effective in inducing remission in CD, increasing *Bifidobacteria* and *Faecalibacterium prausnitzii*.^26^ The combination of metronidazole and ciprofloxacin improves perianal disease symptoms,^27^ while metronidazole and ornidazole successfully prevent clinical postoperative recurrence of CD.^28^ However, antibiotics have side effects on patients and impact the gut microbiota, such as long‐term high‐dose metronidazole causing gastrointestinal disturbances, peripheral neuropathy, and encephalopathy.^29^ Therefore, the use of antibiotics is limited in IBD patients.^30^

Reports on DSS are contradictory. Some studies suggest DSS colitis develops normally without bacteria and may be a more potent colitogenic stimulus, while others report a weaker inflammatory response in germ‐free (GF) conditions, suggesting DSS colitis may be more responsive to antibiotic treatment. The reasons for these discrepancies remain unclear. Our results indicate that GF mice treated with DSS show reduced inflammatory changes in the colon.

DP7 is developed from the identified peptide HH2, exhibits potent antimicrobial activity against several clinically resistant organisms, and the probability of DP7 therapy resulting in the emergence of resistant bacteria and biofilm infections is exceedingly negligible.^12^, ^16^ Our experimental findings show that DP7 has potential as an alternative antibiotic for treating IBD.

DSS‐induced colitis models are highly favored in research into IBD because of their simplicity and reproducibility. Giving mice DSS through their drinking water causes colitis, resulting in weight loss, hemorrhagic diarrhea, ulcer formation, epithelial cell loss, and neutrophilic infiltration. These symptoms are similar to the features of human UC.^31^ Many studies have examined the impact of the gut microbiome on UC development in this mouse model.^32^, ^33^, ^34^, ^35^ Ecological dysregulation of the gut microbiota is associated with intestinal diseases,^36^, ^37^, ^38^, ^39^ suggesting that therapeutic strategies aimed at modulating the gut microbiota should be focused on.^40^, ^41^ Clearing symbiotic *Bacteroidetes* species with antibiotics is known to be effective in preventing the development of colitis in DSS‐treated mice.^42^, ^43^ These previous findings support our results indicating that mice with high *Bacteroidetes* abundance are highly susceptible to DSS. Experimental colitis models^35^, ^44^ and patients with IBD^45^, ^46^ have increased intestinal bacterial diversity, potentially caused by further bacterial infections, which may perpetuate intestinal inflammation.^47^ Choo et al. reported a decrease in *Lachnospiraceae* in mice that received vancomycin treatment,^48^ indicating a potential significant correlation with inflammatory conditions. Further research on how these commensal bacteria establish their ecological niche in the gut will aid in understanding the mechanisms that alleviate the ecological dysregulation linked to IBD.

It is widely acknowledged that commensal bacteria play a role in regulating intestinal epithelial cell turnover, promoting epithelial repair, and reorganizing TJs, all of which are essential for improving barrier function. DSS initially causes crypt loss and epithelial thinning, followed by inflammatory cell infiltration.^17^ Maintaining epithelial homeostasis early in the disease may reduce inflammation and mitigate disease severity. Our findings demonstrate that DP7 promotes colon epithelial healing in disrupted monolayers, aligning with modern UC treatment strategies, where mucosal healing is gaining attention as a key therapeutic endpoint in IBD clinical trials. Therefore, microbial dysbiosis could trigger pathological responses. When the intestinal epithelial mucosa is damaged, microbial translocation disrupts bacterial homeostasis, leading to the production of toxic substances that promote colitis. Repairing the intestinal mucosal barrier can alleviate microbiota disorders, making the integrity of the barrier a key intervention target for colitis treatment. Our results show that DP7 effectively restored the colonic mucosal barrier postcolitis, primarily by significantly upregulating ZO1, claudin, and occludin expression. Additionally, 16S sequencing indicated that the microbiota of DP7‐treated mice differed significantly from that of DSS model mice, revealing that DP7 modulates gut microbiota composition. More specifically, DP7 supplementation significantly increased the prevalence of *Muribaculaceae*, a bacterium that utilizes mucin, which significantly increased after DP7 treatment, indicating a positive correlation with body weight and colon length. Numerous previous studies have reported favorable outcomes of *Muribaculaceae* in safeguarding gut health.^49^, ^50^ It suppresses the expression of proinflammatory cytokines, potentially accounting for our experimental findings that the levels of IL‐1β and IL‐6 expression were reduced in the DP7 group. Moreover, we observed alterations in the microbiota structure and altered α diversity in the DSS group, and although the changes in the microbial community were most likely caused by severe inflammation, it cannot be excluded that the changes were also due to direct or indirect effects of DSS itself. For example, DSS increases the permeability of intestinal mucus and the penetration of bacteria into epithelial tissue, which could create new ecological niches for bacteria before inflammation occurs.^35^ In the future, we will select suitable probiotics or bacteria‐targeting drugs to enhance DP7's efficacy.

The gastrointestinal tract is a mucosal surface constantly exposed to foreign antigens and microorganisms, protected by various immunologically active structures and cells. Epithelial cells play a direct role in immune supervision and host response direction in the gut, expressing multiple pattern recognition receptors. They can also produce chemokines for bone marrow and lymphocytes following exposure to inflammatory stimuli. A population of innate lymphocytes in the gut can be activated and produce effector cytokines upon stimulation. These lymphocytes can play both protective and pathogenic roles in the inflammatory process. The composition and metabolic activity of the intestinal microbial community produce short‐chain fatty acids, polysaccharides, and other substances, which have profound effects on mucosal immune regulation. For example, polysaccharides facilitate the transformation of CD4^+^ T cells into regulatory T cells in a TLR2‐dependent manner; this process inhibits IL‐17 production, successfully preventing a major proportion of inflammatory damage.^51^, ^52^, ^53^, ^54^ These preexisting findings may explain the Th1 and Th17 cell changes we detected.

Mice with colitis treated with DP7 presented a reduction in the number of Th1 and Th17 cells in the gut, resulting in the control of inflammation. Additionally, the proportion of Treg cells was restored, which further regulated immune homeostasis in the gut, contributing to the treatment and remission of colitis. However, in normal individuals, the effects of DP7 on the immune system are not completely uniform. In normal mice, injection of DP7 resulted in a slight increase in the number of Th1 cells, while the immunomodulatory function of DP7 had a more significant effect on intestinal immune homeostasis than did systemic immune regulation. In mice with colitis, DP7 not only restored immune homeostasis in the intestine but also helped Th cells in the spleen restore homeostasis. These findings demonstrate the immunomodulatory function of DP7. Furthermore, our findings demonstrated that the immunomodulatory function of DP7 appeared to be influenced by the intestinal flora, as evidenced by the observation that the injection of DP7 did not result in alterations in Th cells in vivo following the removal of the intestinal flora. These findings further support the notion that the gut flora affects immunity.

DP7 diminishes the inflammatory response induced by DSS, and its mechanism of action is likely associated with ameliorating the inflammatory state and sustaining the integrity of the epithelial barrier of the intestine. More importantly, the ability of DP7 to reduce inflammation may be facilitated by modulating the intestinal microbiota. Many studies have confirmed the importance of the gut microbiota in intestinal inflammatory diseases. Transplantation of the microbiota from mouse models with IBD to healthy mice may induce this disease.^55^, ^56^ Conversely, medications for IBD have the ability to modify or regulate the gut microbiota.^57^, ^58^ The experimental results detailed in this paper suggest that DP7 could be a viable therapeutic option for noninfectious intestinal inflammation while also providing potential targets for future microbiome research, such as *Muribaculaceae*.

*Muribaculaceae* were enriched in the mouse colon. Subsequent analyses revealed that their abundance positively correlated with colon length and weight, and negatively with DAI, following DP7 injection and FMT from DP7‐administered mice. Due to experimental limitations, we did not conduct clinical trials; instead, we reviewed literature and databases on gut microbiota in clinical patients, uncovering studies on *Muribaculaceae*. From Peryton, we found that metagenomic analysis of fecal samples from 40 healthy volunteers and 32 colorectal cancer (CRC) patients showed a significant decrease in *Muribaculaceae* in CRC patients (0.610%) compared to healthy controls (0.491%). Additionally, gutMDisorder reported that 16S rRNA sequencing of stool samples from 24 polycystic ovary syndrome (PCOS) patients revealed a complete absence of *Muribaculaceae*, contrasted with 1.1473% in healthy controls. These findings suggest that *Muribaculaceae* may have a protective role against inflammatory diseases. Further research is necessary to elucidate their specific mechanisms in human health and disease. We acknowledge that these clinical data are limited and further research is needed to validate these findings. However, we believe that these findings provide preliminary evidence supporting a potential link between *Muribaculaceae* abundance and human health. We are currently working to expand our research to explore the clinical implications of *Muribaculaceae* in human disease and to investigate the potential of utilizing this bacterial family as a therapeutic target. It is important to note that *Muribaculaceae* is a complex bacterial group, and it is possible that specific species or strains within this family are responsible for the observed effects, rather than the entire family. Further research, including bacterial isolation and culturing techniques, is necessary to identify these specific bacteria and gain a more detailed understanding of their roles in health and disease.

Importantly, this study merely observed microbiota changes without demonstrating whether these changes stemmed from drug treatment or were responsible for colitis remission; therefore, further investigation is needed to determine the underlying mechanisms involved.

## 4 MATERIALS AND METHODS

### 4.1 Preparation of DP7

Shanghai Science Peptide Biological Technology synthesized DP7 (VQWRIRVAVIRK) and purified by high‐performance liquid chromatography to 99% purity.^13^

### 4.2 Animals

Six‐week‐old male C57BL/6 mice were obtained from Beijing Vital River Laboratory Animal Technology Co., Ltd. and housed under pathogen‐free conditions with a 12‐h light/dark cycle at 22 ± 2°C.

### 4.3 DSS‐induced colitis, antibiotic treatment, DP7 treatment, and FMT

Mice were administered 2%–4% DSS (40,000–50,000 Da, MP Biomedicals) in drinking water for 7 days, followed by tap water for 3 days.^59^ Body weight was monitored daily, and the DAI was calculated as previously described.^31^ At the end of the experiment, intestinal tissues were collected for colitis and regeneration assessment.

To deplete gut microbiota, mice received broad‐spectrum antibiotics (streptomycin, ampicillin, and colistin) via drinking water for 7 days.^60^ Microbiota depletion was confirmed by culturing stool samples on Lysogeny Broth (LB) agar plates under anaerobic and aerobic conditions. DSS‐induced colitis was then initiated.

Fecal transplants from donor mice in DSS and DP7 groups were prepared at 8 days post‐treatment by suspending feces in phosphate‐buffered saline (PBS; 100 mg/mL), centrifuging, and administering 200 µL of the supernatant to each antibiotic‐treated mouse via oral gavage daily. Fresh transplants were used within 10 min.

Alternatively, mice were treated with DP7 (0.5 mg/kg, i.v., every 2 days^12^, ^14^, ^16^; controls received PBS).

### 4.4 Tissue harvesting and processing

The mice were killed by cervical dislocation at the end of the experiment, and then, samples of tissue and serum were collected. The serum, colon, and colon contents were harvested and immediately frozen at −80°C. The tissues were fixed in 4% paraformaldehyde.

#### 4.4.1 RNA extraction and quantitative real‐time PCR

Total RNA was extracted from homogenized tissue using a Foregene kit. Cytokine transcription levels were analyzed with SYBR Green PCR Master Mix (Vazyme) on a CFX96 real‐time system (Bio‐Rad).

#### 4.4.2 Histological analyses

Tissues were sectioned (4 µm), and stained with H&E and AB. For IF, sections were incubated overnight at 4°C with primary antibodies, followed by Alexa Fluor 555 secondary antibody (1:200) and 4',6‐Diamidino‐2‐phenylindole (DAPI) counterstaining. Terminal dUTP Nick End Labeling (TUNEL) staining was done per the kit protocol.

#### 4.4.3 ELISA

The following indicators were tested: the serum concentrations of IL‐6, IL‐1β, and IL‐10. All operations were performed in strict accordance with the kit instructions (Thermo Scientific).

#### 4.4.4 Flow cytometry experiments

Monoclonal antibodies for CD45, CD3, CD4, CD25, Foxp3, IFN‐γ, IL‐17A, and FVS700 (BD Biosciences) were used. Flow cytometry (FACS Calibur; BD Biosciences) was used to obtain the number of cell events, and the data were analyzed with NovoExpress software.

### 4.5 16S rRNA analyses of the fecal microbiota

16S rDNA sequencing was conducted by Beijing Novogene Technology Co., Ltd.^61^ Raw tags were quality filtered using QIIME2, and DADA2 was employed to generate ASVs with 100% similarity.

### 4.6 Statistical analysis

Data were analyzed using GraphPad Prism 9 and are shown as mean ± standard error of the mean (SEM). Statistical significance (*p* < 0.05) was assessed via one‐way analysis of variance (ANOVA) or *t*‐test. *p < 0.05, **p < 0.01, ***p < 0.001, ****p < 0.0001.

## AUTHOR CONTRIBUTIONS

Li Yang and Binyan Zhao designed the study and wrote the manuscript. Binyan Zhao and Hongyou Zhou conducted the experiments and collected the data. Ke Lin, Jie Xu, Bailing Zhou, Daoyuan Xie, Jing Ma, Lei Yang, and Chunyan Su analyzed the data and prepared the figures. Li Yang reviewed the manuscript and supervised the study. All the authors have read and approved the final manuscript.

## CONFLICT OF INTEREST STATEMENT

The authors declare no conflicts of interest.

## ETHICS STATEMENT

All animal procedures followed the guidelines approved by the Animal Protection Committee of Sichuan University (Sichuan, China; ID: 20230307029).

## Supporting information



Supporting Information

## Data Availability

Sequencing data have been submitted to the NCBI Sequence Read Archive under accession No. PRJNA1171752.
