# Supplementary material for: Antimicrobial peptide DP7 alleviates dextran sulfate sodium (DSS)‐induced colitis via modifying gut microbiota and regulating intestinal barrier function
Source: MedComm (2020). 2025 Jan 30;6(2):e70085. doi: 10.1002/mco2.70085 (PMC11782841; doi:10.1002/mco2.70085)
Supplement: Supplementary file 1 — Supporting Information [file MCO2-6-e70085-s001.docx]

Antimicrobial peptide DP7 alleviates Dextran Sulfate Sodium (DSS)-induced colitis via modifying gut microbiota and regulating intestinal barrier function

**Binyan Zhao^1, #^, Hongyou Zhou^1, #^, Ke Lin^1^, Jie Xu**^1^, **Bailing Zhou** ^1^**, Daoyuan Xie**^1^**, Jing Ma^2^, Lei Yang^2^, Chunyan Su^2^, Li Yang**^*^

^1^ Department of Biotherapy, Cancer Center and State Key Laboratory of Biotherapy, West China Hospital, Sichuan University, Chengdu, 610041, China.

^2^ Biological Products Inspection Institute of Sichuan Institute of Drug Inspection, The People’s Republic of China. Chengdu, 610041, China.

^#^ Binyan Zhao and **Hongyou Zhou** contributed equally to this work

* Correspondence:

Prof. Li Yang, Department of Biotherapy，Cancer Center and State Key Laboratory of Biotherapy，West China Hospital, Sichuan University, Chengdu, 610041, China; Tel.: 18628182400, Email: [yl.tracy73@gmail.com](mailto:yl.tracy73@gmail.com)


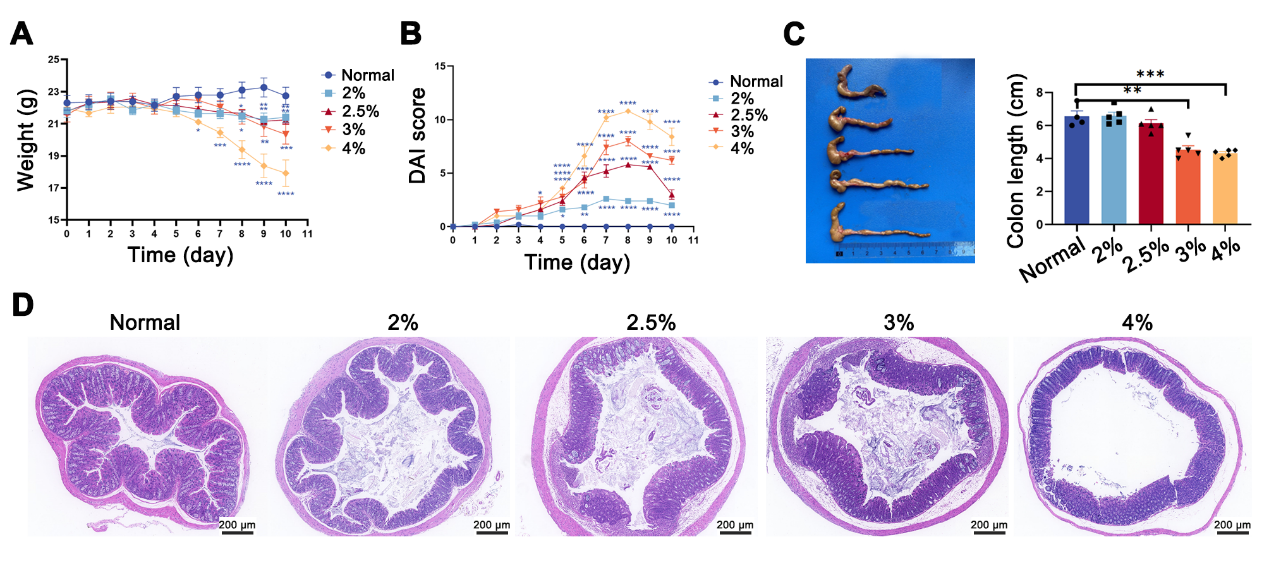


Figure S1. DSS-induced colitis model. To determine the optimal modelling conditions, the mice were provided varying concentrations of DSS-water for 7 days. The rats were subsequently given regular drinking water for 3 days to recover while being monitored for (A) changes in body weight and (B) DAI scores. At the end of the experiment, (C) the colon was extracted from each mouse, and its length was measured. (D) Mouse colon was analysed via H&E staining. Scale bars are 200 μm. (n = 5 per group)


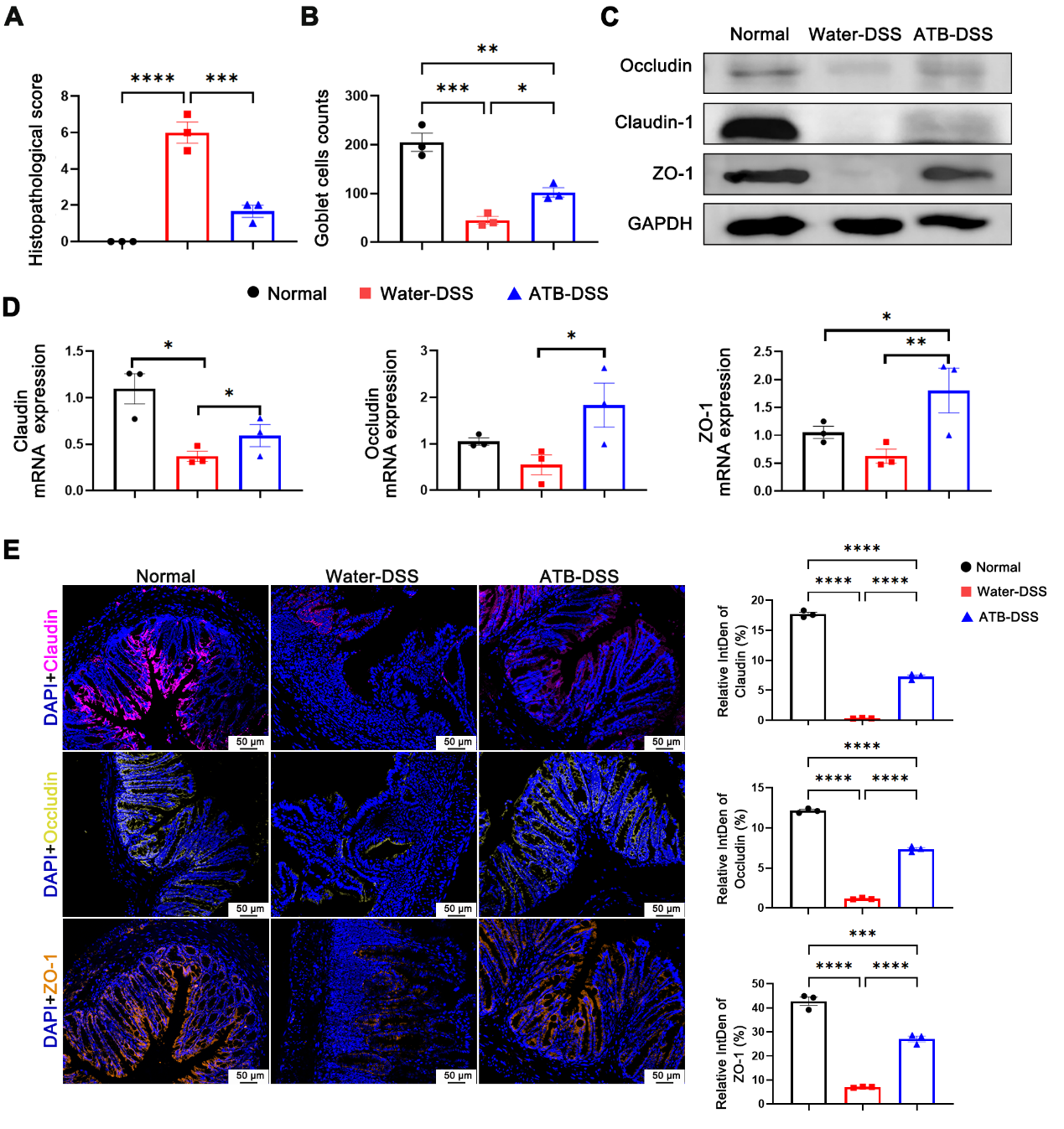


Figure S2. The administration of antibiotics resulted in a reduction in mucosal damage. (A)The histological scoring based on H&E staining results, and (B) The quantification of goblet cell numbers. (C)Western blot analysis of colons was performed with anti-Occludin, anti-Claudin-1, anti-ZO-1, and anti-GAPDH antibodies. (D) The mRNA expression levels of Claudin-1, Occludin and ZO-1 in the colon were detected via RT‒PCR. (E) claudin-1, Occludin and ZO-1 expression was determined via confocal immunofluorescence. The data on the far right represent the quantified results of immunofluorescence intensity. Scale bars are 50 μm. (n = 3 per group)


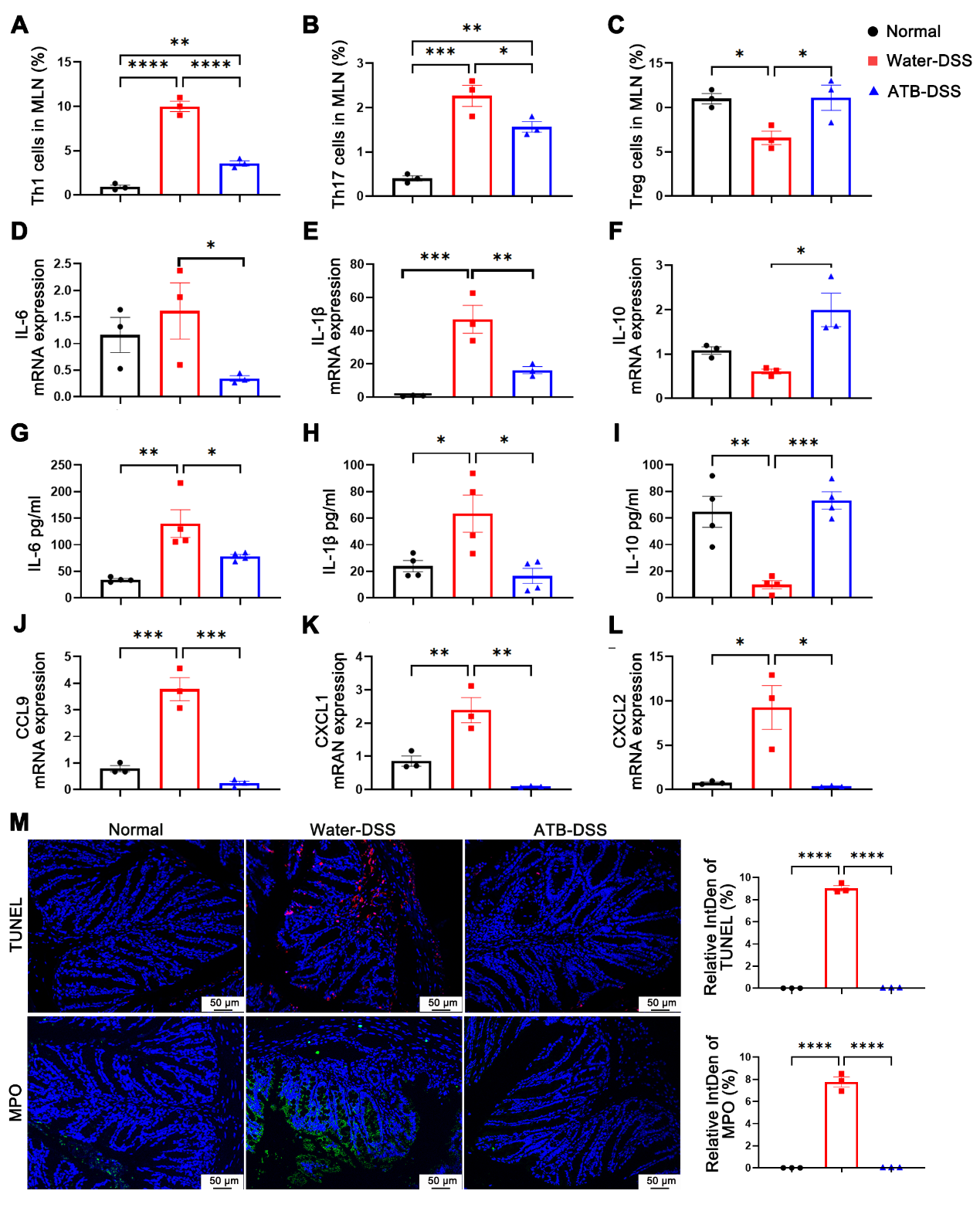


Figure S3. The administration of antibiotics resulted in the relief of colonic inflammation. (A-C) Changes in the proportions of Th1, Th17 and Treg cells in mesenteric lymph nodes were identified via flow cytometry. (D-F) The mRNA expression levels of IL‐6, IL‐1β and IL-10 were detected in the colon via RT‒PCR. (G-I) The concentrations of IL‐6, IL‐1β and IL-10 in the serum. (J-L) The mRNA expression levels of CCL9, CXCL1 and CXCL2 in the colon were quantified via RT‒PCR. (M) Representative fluorescence images of TUNEL staining of colonic sections (top) and MPO immunofluorescence staining (bottom). The data on the far right depict the quantified immunofluorescence intensity results. Scale bars are 50 μm. (n = 3 per group)


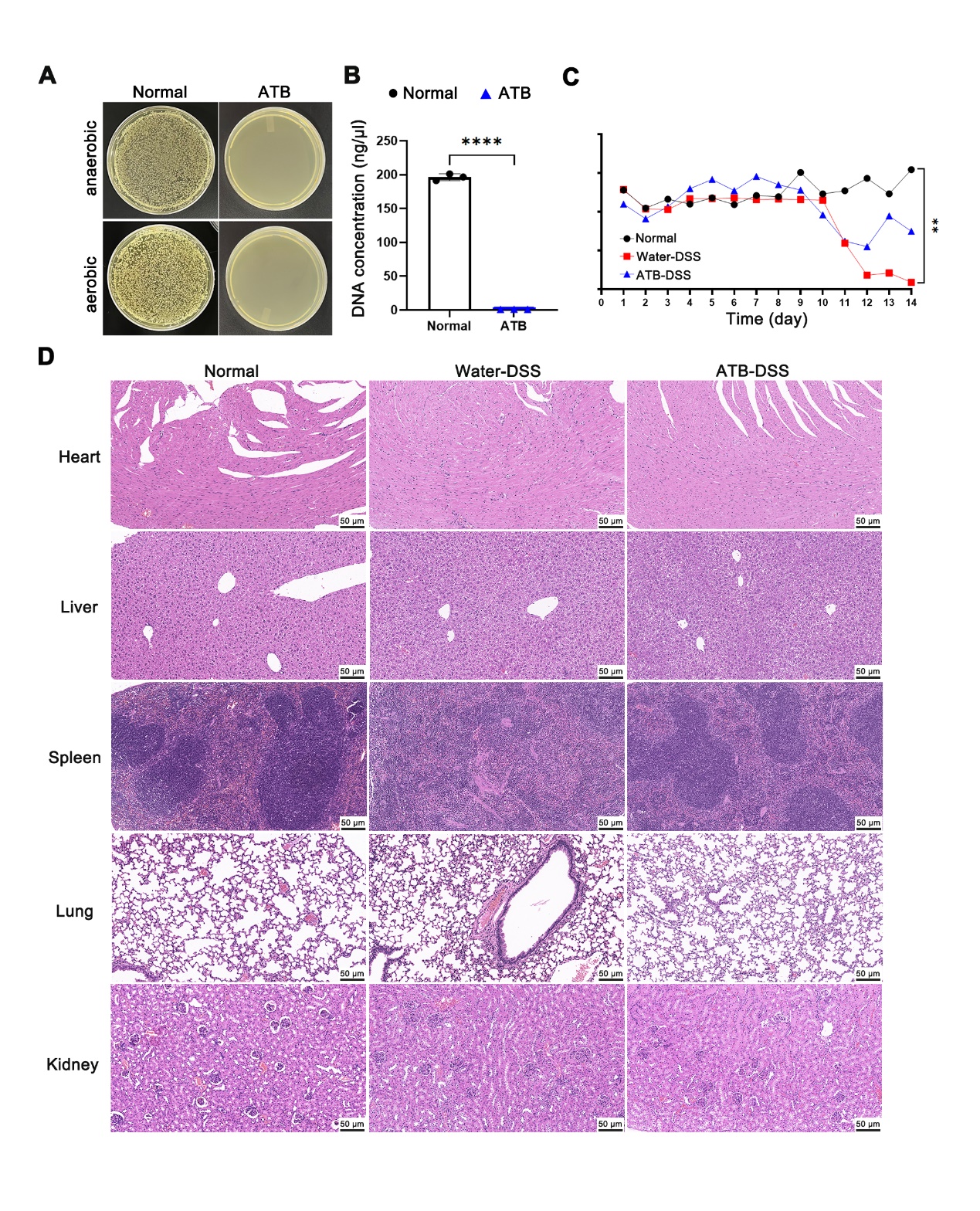


Figure S4. Faecal samples (100 mg) were collected and mixed with 1 ml of sterile water, and the supernatants were collected for bacterial culture. The results of the bacterial culture, which was conducted under aerobic and anaerobic conditions, are presented in (A) a representative picture. No bacterial community grew on the plate following antibiotic treatment. (B) Determination of the bacterial DNA concentration. After antibiotic treatment, the concentration of faecal DNA decreased significantly. These data show that antibiotic treatment effectively eliminated a significant portion of the gut microbiome. (C) Food consumption measurements in mice. (D) Microscopic examination of tissue sections stained with hematoxylin and eosin revealed no evidence of significant histopathological alterations in the major organs. Scale bars are 50 μm. (n = 3 per group)

To investigate the mitigating effect of DP7 on DSS-induced colitis, mice were treated with a 4% aqueous solution of DSS for 7 days to induce experimental colitis, followed by intravenous administration of DP7 at the optimal dose previously reported^16–18^. To determine the most effective timing of administration, we evaluated symptoms in mice treated via three different approaches. DP7 injection during DSS administration significantly reduced DSS-induced colitis compared with that in the other groups (Figure S5A-E). Histological analysis revealed that symptoms of inflammatory cell infiltration, mucosal damage and mucin secretion in the colon were attenuated more significantly when DP7 was administered during the modelling process than before or after (Figure S5F-G).


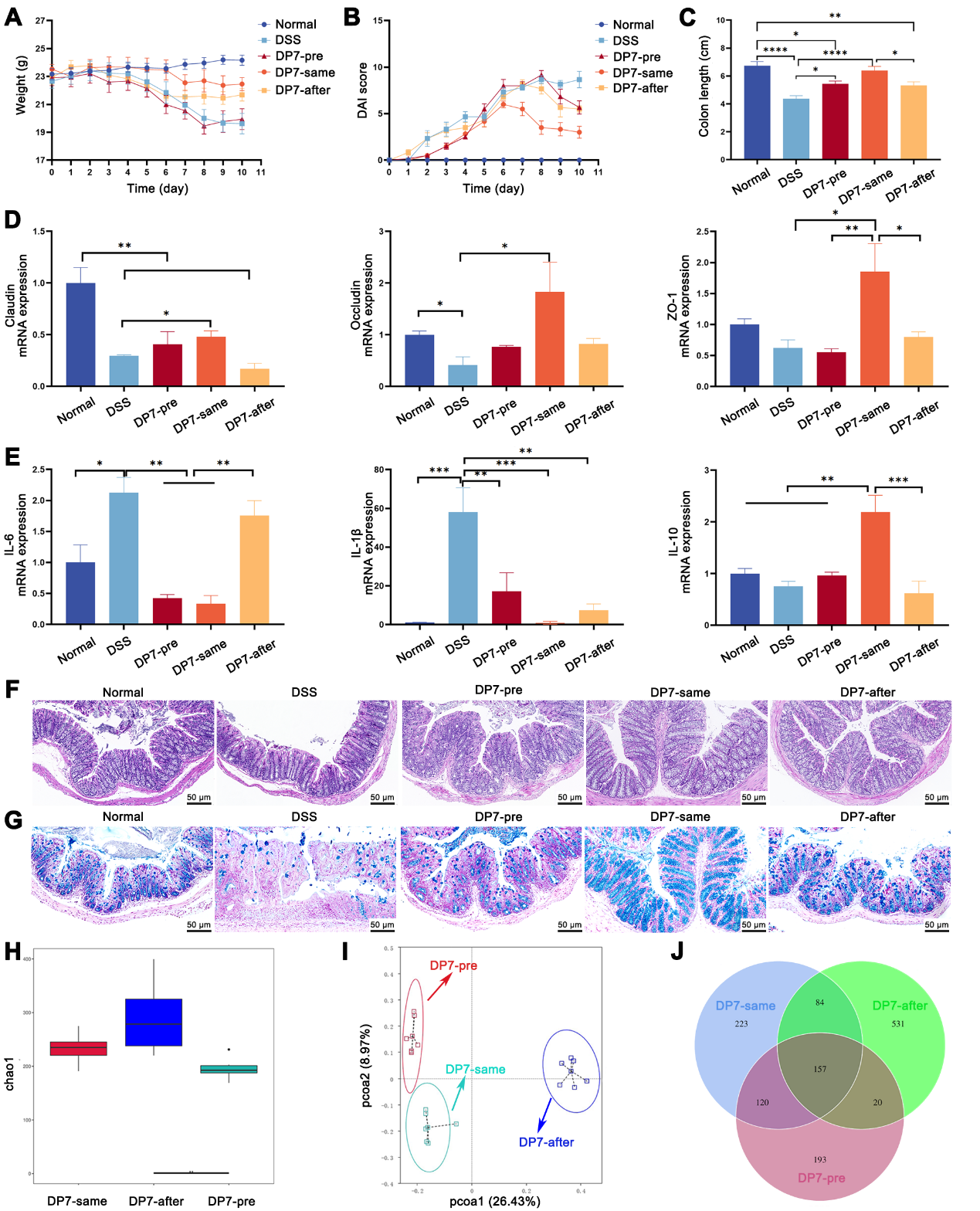


Figure S5. The ideal timing for DP7 administration was explored, and the effects of different schedules on the intestinal flora structure were analysed. The mice were monitored throughout the experiment for changes in (A) body weight and (B) DAI scores. (C) Colon length measurements were taken at the end of the experiment. The mRNA expression levels of (D) Claudin-1, Occludin, and ZO-1 and (E) cytokines in the colon were detected via RT‒PCR. Representative colon images of (F) H&E-stained and (G) Alcian blue-stained samples. (H) The Chao1 index of different DP7-treated groups. (I) The PCoA plot was analysed with PERMANOVA for all three groups. (J) Venn diagrams were constructed to analyse the shared and specific ASVs between various DP7-treated groups. (n = 3 per group)


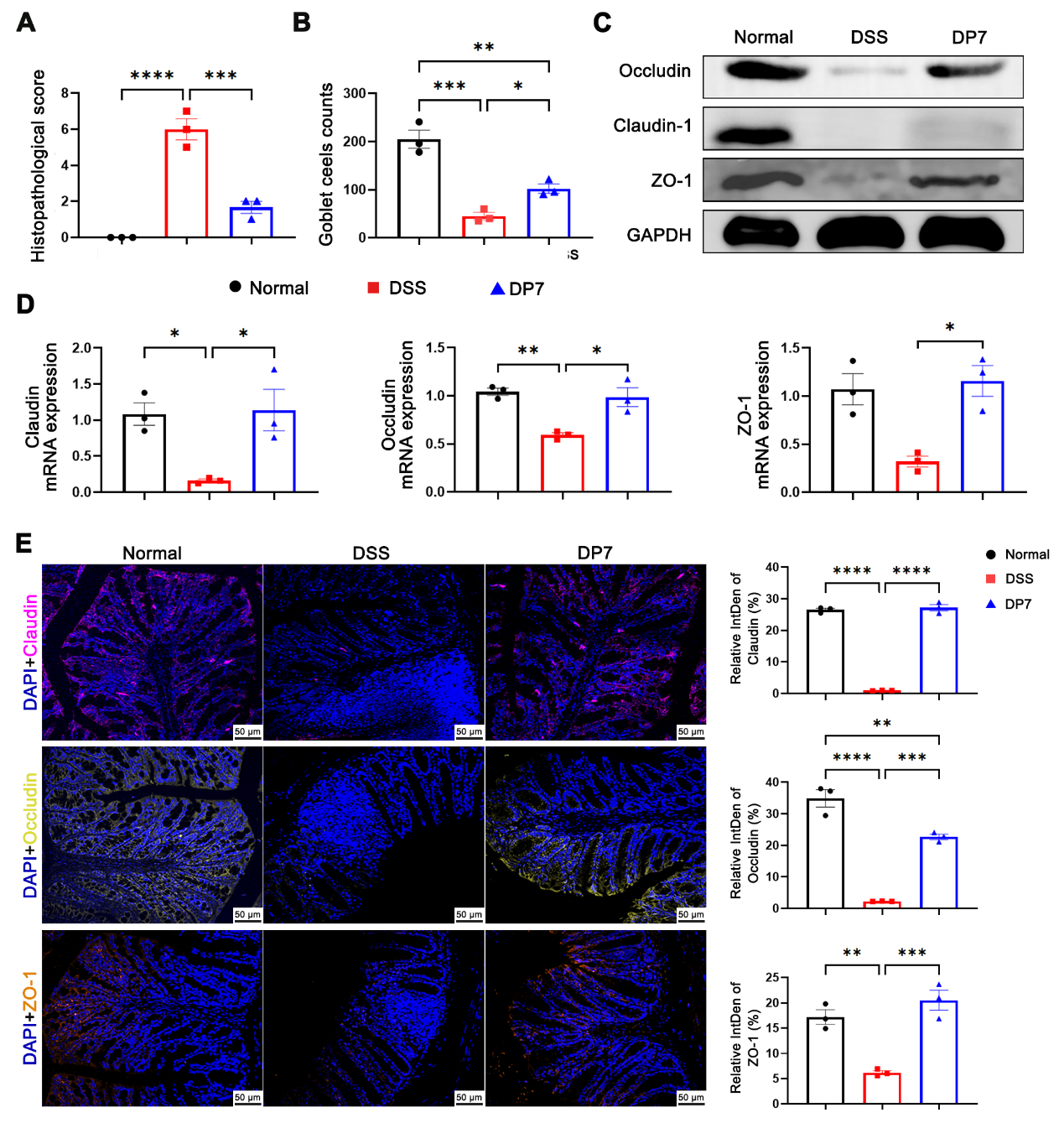


Figure S6. DP7 promoted the repair of mucosal damage. (A) Evaluation of histological scores using H&E staining, and (B) Quantification of goblet cell counts. (C) Western blot analysis of colons was performed with anti-Occludin, anti-Claudin-1, anti-ZO-1, and anti-GAPDH antibodies. (D) The mRNA expression levels of Occludin, Claudin-1 and ZO-1 in the colon were detected via RT‒PCR. (E) Claudin-1, Occludin and ZO-1 expression was determined via confocal immunofluorescence. Quantified immunofluorescence intensity results are shown on the far right. Scale bars are 50 μm. (n = 3 per group)


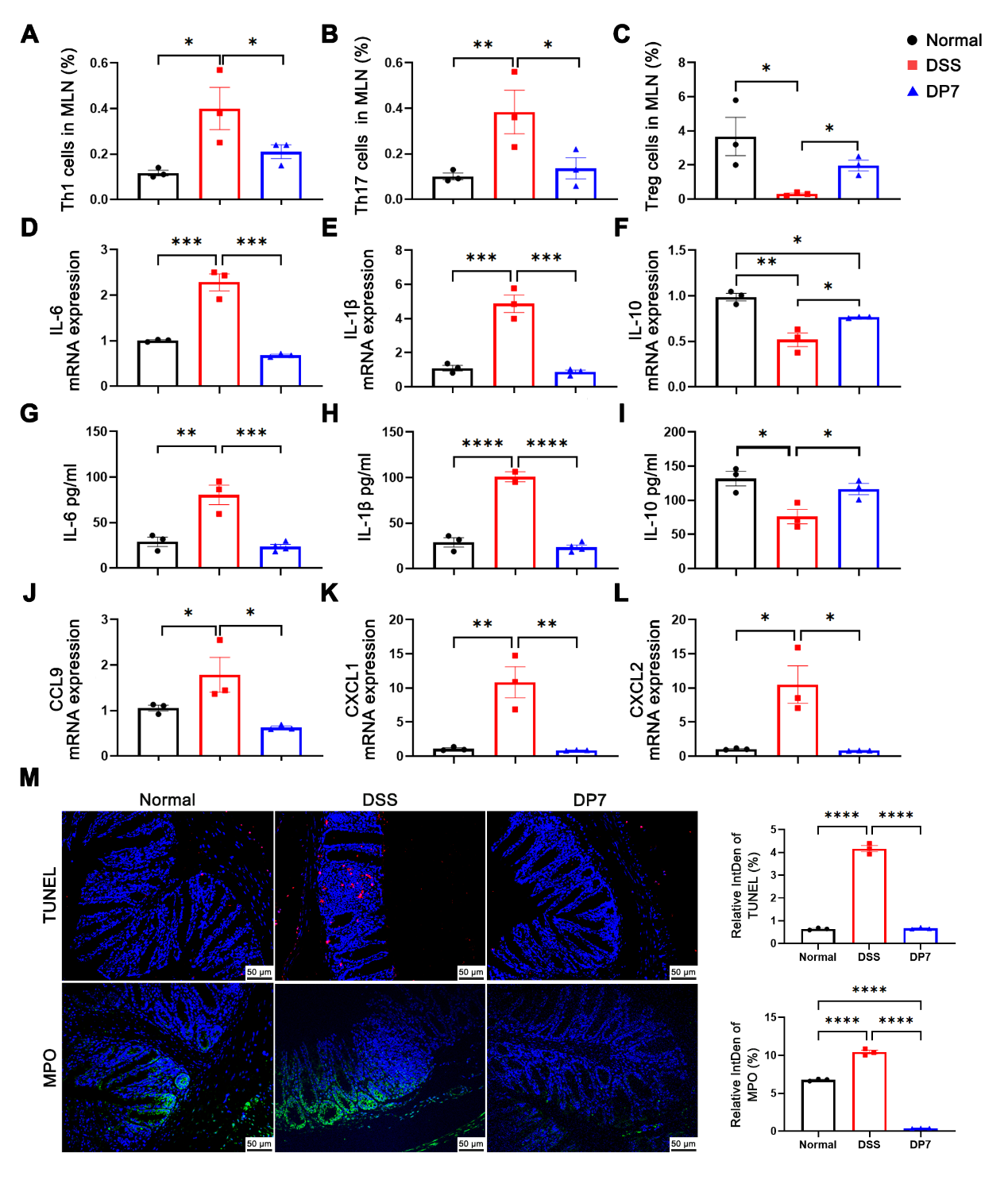


Figure S7. DP7 administration resulted in a reduction in colonic inflammation. (A-C) Changes in the proportions of Th1, Th17 and regulatory T (Treg) cells in mesenteric lymph nodes were identified via flow cytometry. (D-F) The mRNA expression levels of IL‐6, IL‐1β and IL-10 were detected in the colon via RT‒PCR. (G-I) The concentrations of IL‐6, IL‐1β and IL-10 in the serum. (J-L) The mRNA expression levels of CCL9, CXCL1 and CXCL2 in the colon were quantified via RT‒PCR. (M) Representative fluorescence images of TUNEL staining of colonic sections (top) and MPO immunofluorescence staining (bottom). The far-right panel presents the quantified results of immunofluorescence intensity. Scale bars are 50 μm. (n = 3 per group)


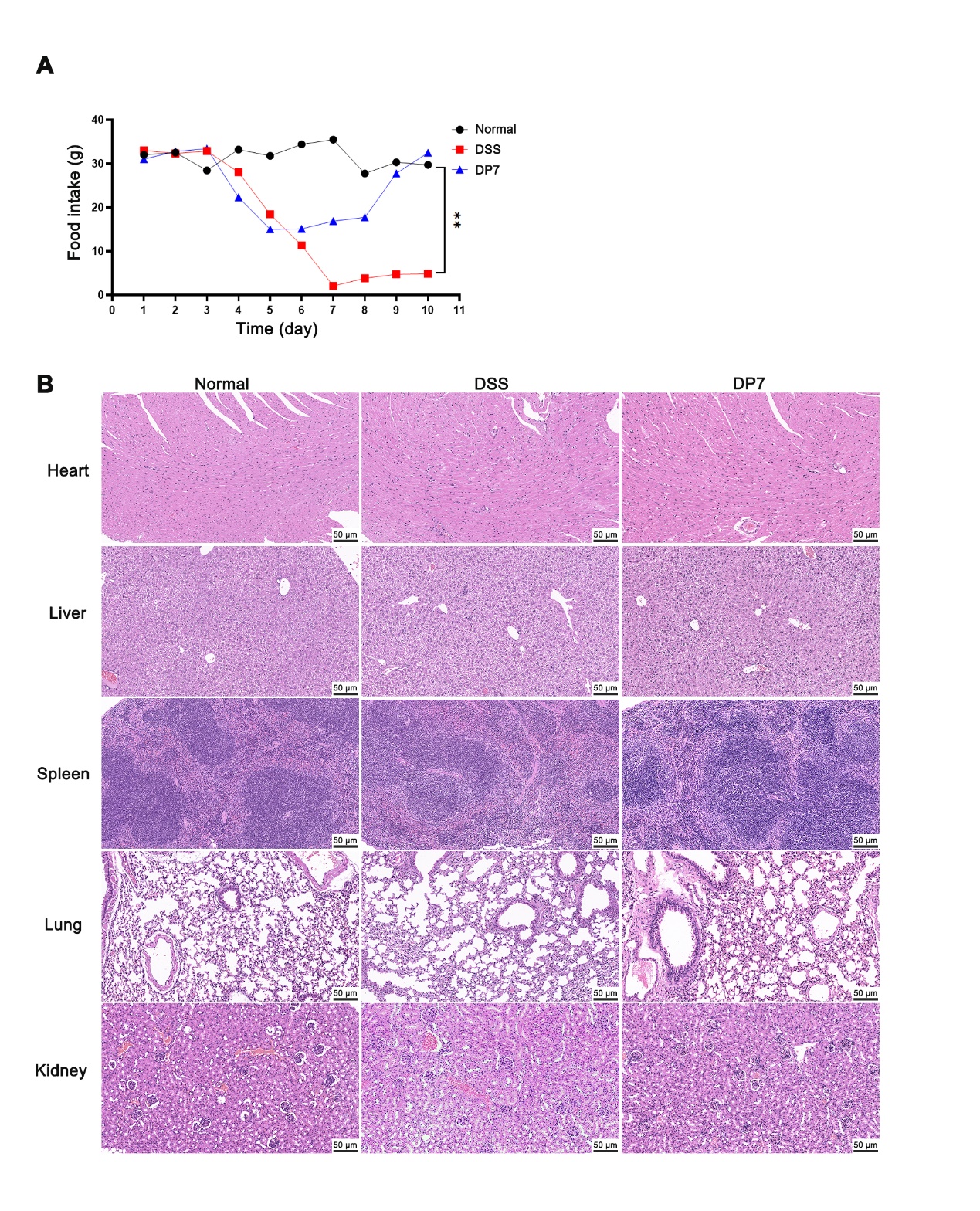


Figure S8. (A) Food intake monitoring of the mice. (B) Histopathological examination of the liver, spleen, kidney, lung, and heart of mice after DP7 administration revealed no significant pathological changes. This preliminary assessment suggests the drug's safety profile. Scale bars are 50 μm. (n = 6 per group)


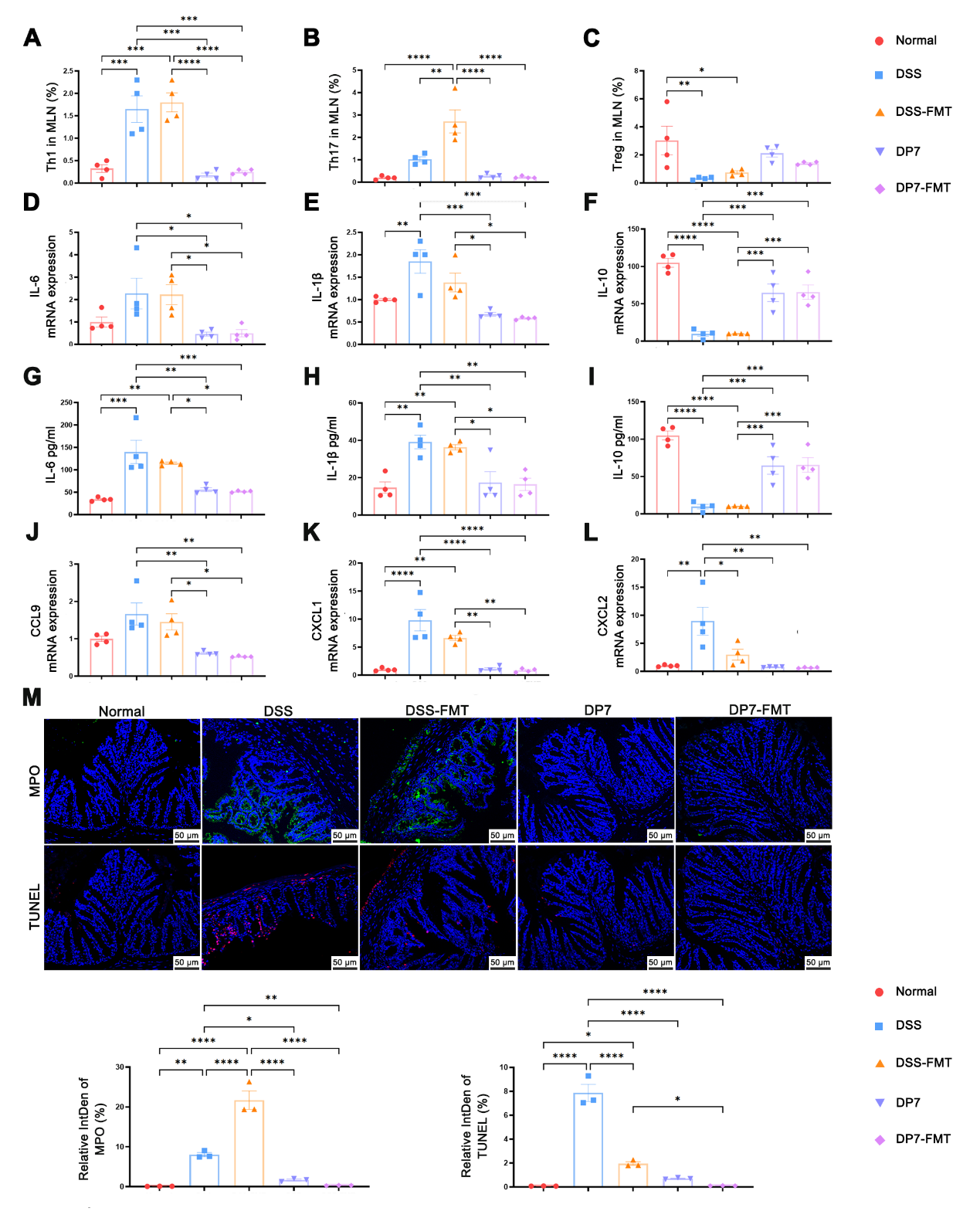


Figure S9. The administration of microbiota from DP7-treated mice resulted in a relief of colonic inflammation. (A-C) Changes in the proportions of Th1, Th17 and regulatory T (Treg) cells in mesenteric lymph nodes were identified via flow cytometry. (D-F) The mRNA expression levels of IL‐6, IL‐1β and IL-10 were detected in the colon via RT‒PCR. (G-I) The concentrations of IL‐6, IL‐1β and IL-10 in the serum. (J-L) The mRNA expression levels of CCL9, CXCL1 and CXCL2 in the colon were quantified via RT‒PCR. (M) Representative fluorescence images of MPO immunofluorescence staining (top) and TUNEL staining of colonic sections (bottom). The data below the image depict the quantified immunofluorescence intensity results. Scale bars are 50 μm.


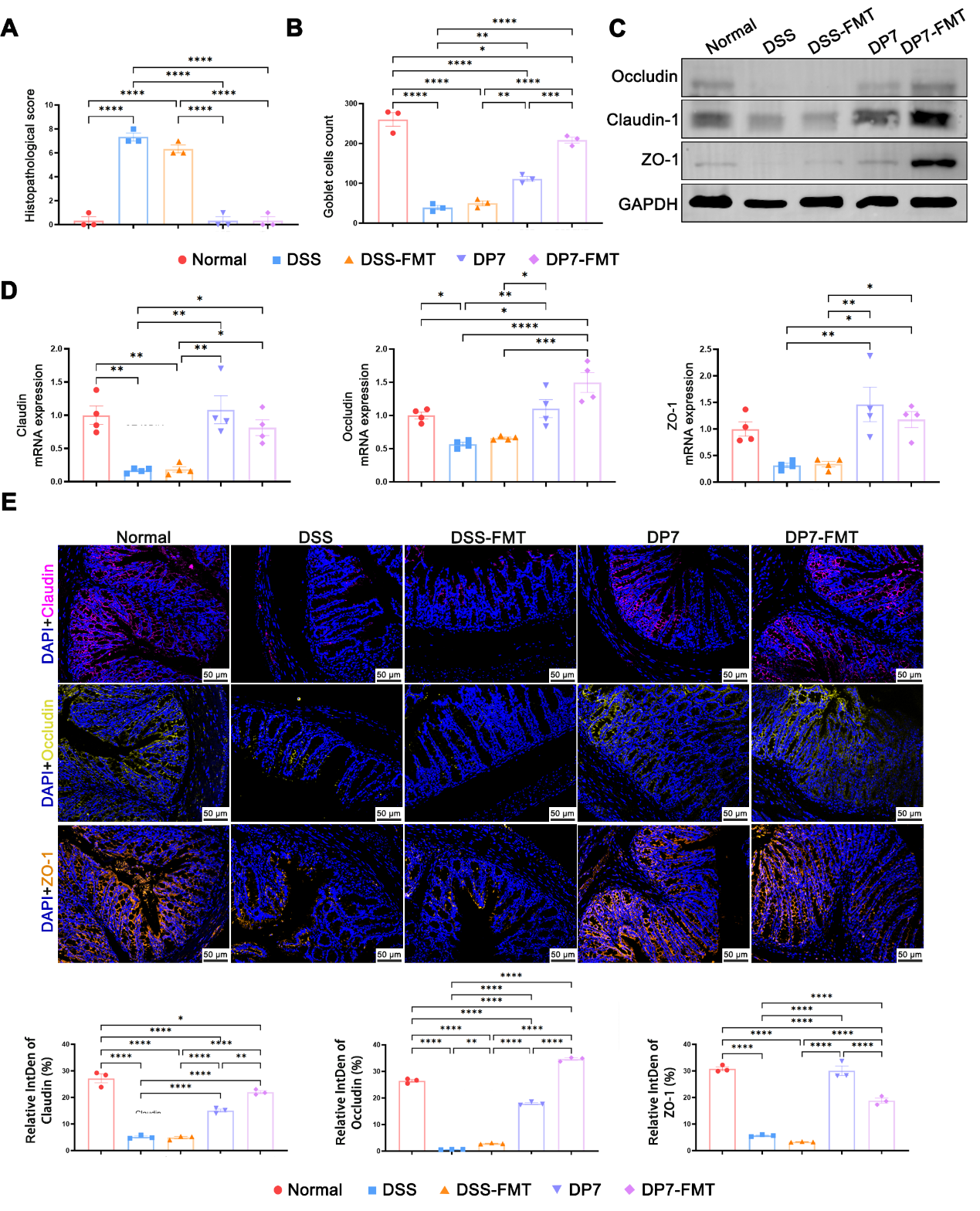


Figure S10. The transplantation of microbiota from mice treated with DP7 promoted the repair of mucosal damage. (A) The histological assessment using H&E staining outcomes, and (B) The enumeration of goblet cells. (C) Western blot analysis of colons was performed with anti-Occludin, anti-Claudin-1, anti-ZO-1, and anti-GAPDH antibodies. (D) The mRNA expression levels of Claudin-1, Occludin and ZO-1 in the colon were detected via RT‒PCR. (E) Claudin-1, Occludin, and ZO-1 expression was assessed via confocal immunofluorescence, with quantified intensity values shown below the image. Scale bars are 50 μm.


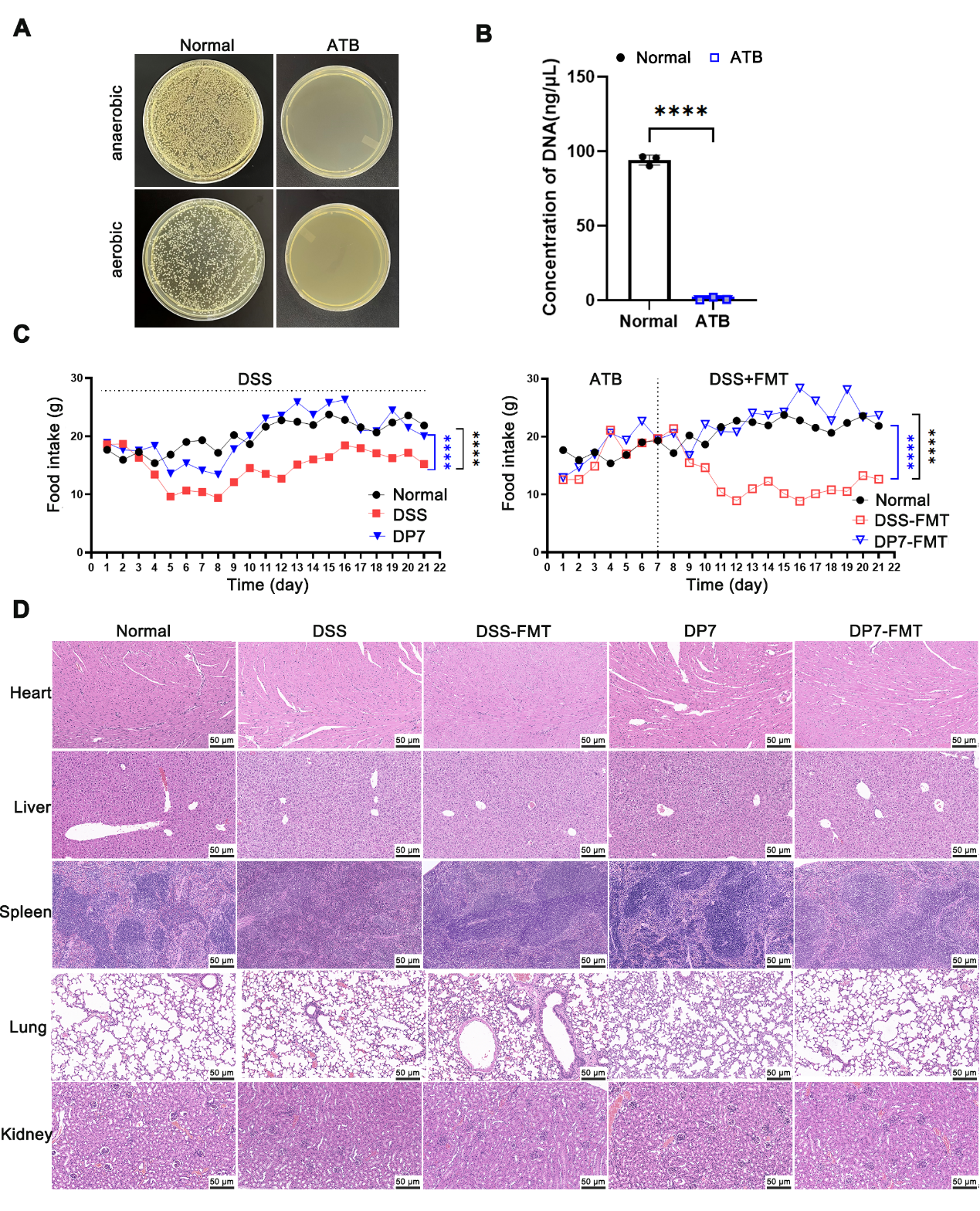


Figure S11. Compared with DSS‐derived faecal microbiota, microbiota transplantation from mice treated with DP7 relieves colitis better. The results of the bacterial culture, which was conducted under aerobic and anaerobic conditions, are presented in (A) a representative picture. No bacterial community grew on the plate following antibiotic treatment. (B) Determination of the bacterial DNA concentration. After antibiotic treatment, the concentration of faecal DNA decreased significantly. These results demonstrated that antibiotics were effective at eradicating the gut microbiota. (C) Food consumption measurements in mice. (n = 6 per group) (D) Histological examination of the major organs (heart, liver, spleen, lung, and kidney) of mice treated with the FMT, using H&E staining, was performed to preliminarily assess the “drug's” safety. The results showed no significant pathological changes in these organs. Scale bars are 50 μm.


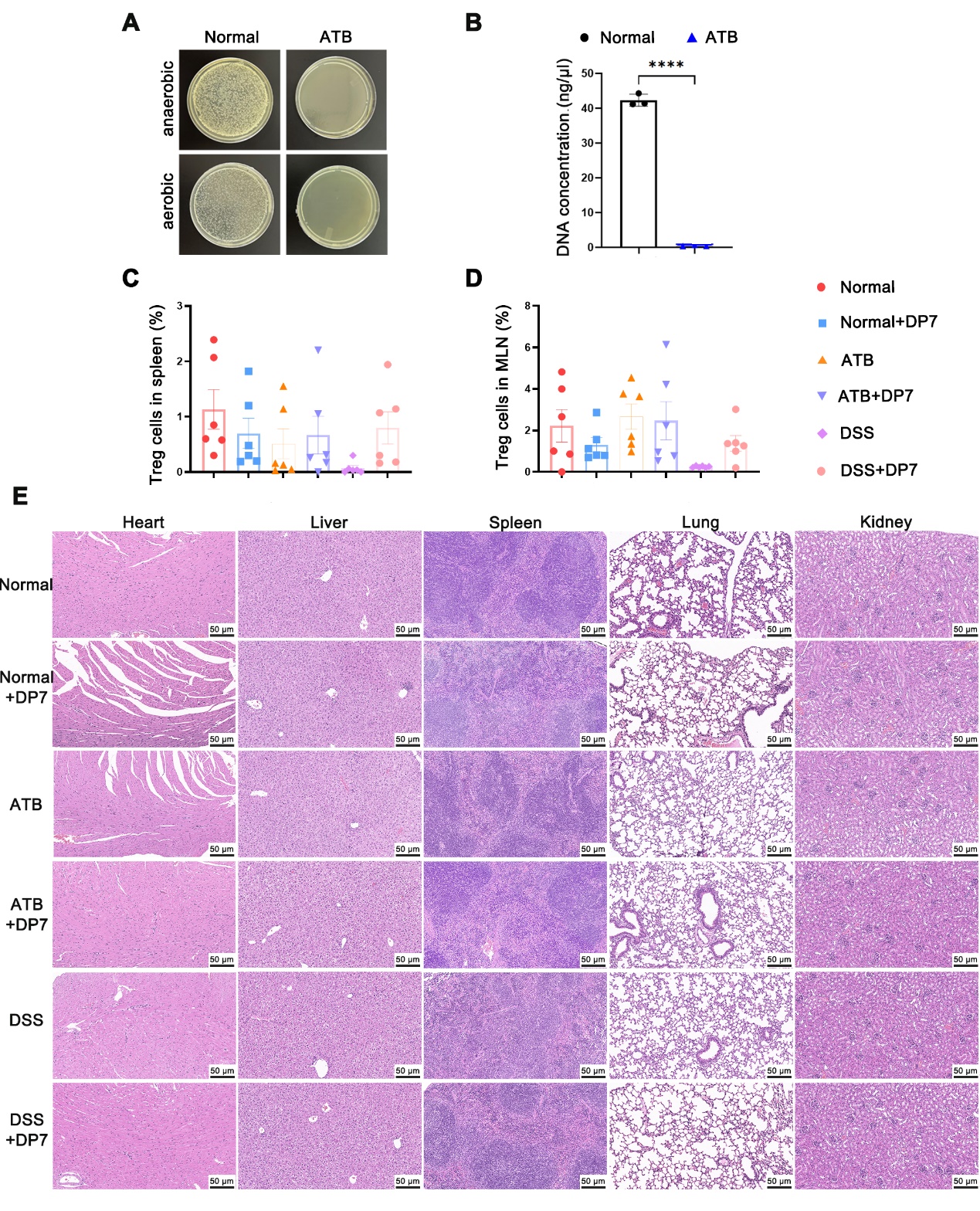


Figure S12. The administration of DP7 was found to affect the immune system of mice.

The results of the bacterial culture, which was conducted under aerobic and anaerobic conditions, are presented in (A) a representative picture. No bacterial community grew on the plate following antibiotic treatment. (B) Determination of the bacterial DNA concentration. After antibiotic treatment, the concentration of faecal DNA decreased significantly. These results demonstrated that antibiotics were effective at eradicating the gut microbiota. Flow cytometry was used to identify changes in the proportions of Tregs in the (C) spleen and (D) mesenteric lymph nodes. (E) The major organs of mice were examined using H&E staining post-treatment to preliminarily assess the safety of DP7. No discernible pathological alterations were observed in the main organs. Scale bars are 50 μm. (n = 3 per group)


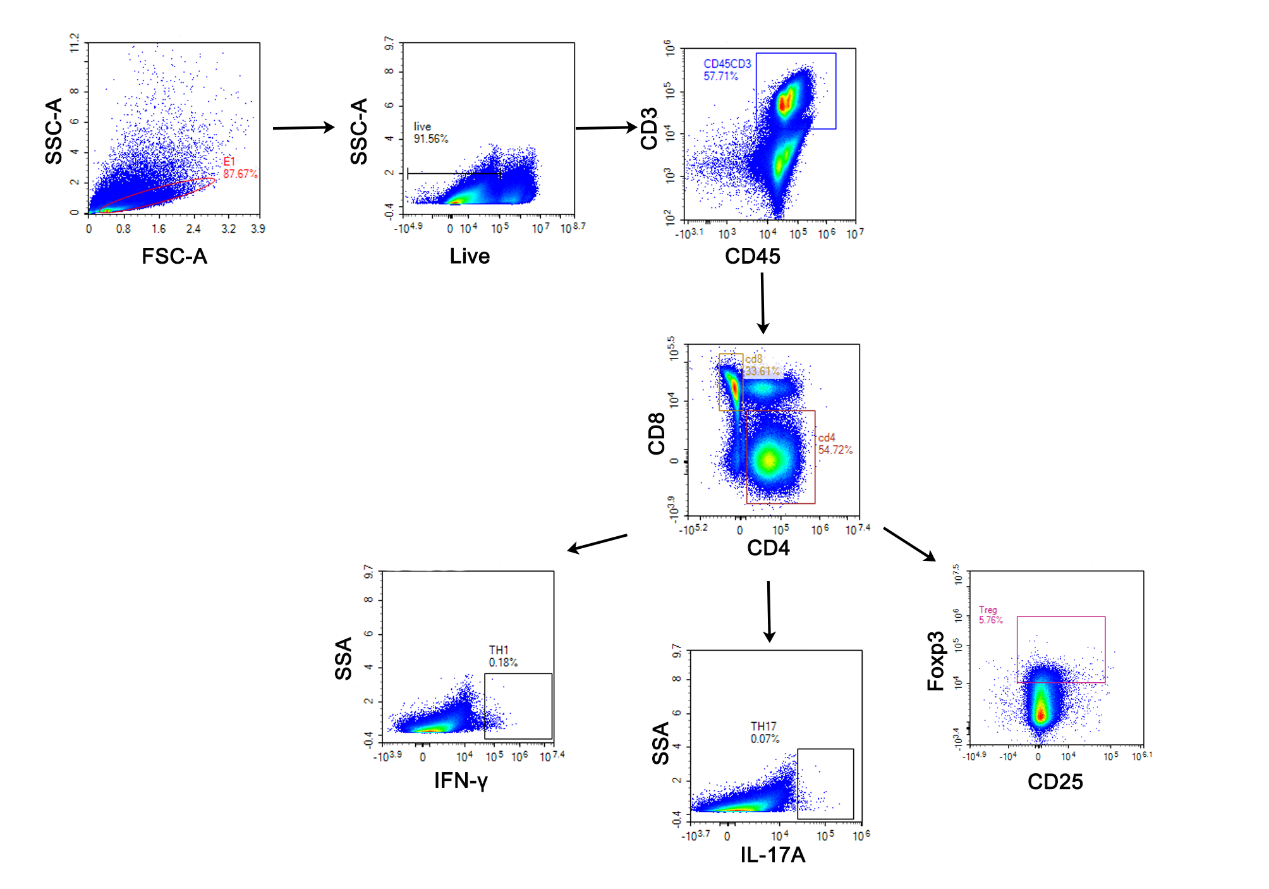


Figure S13. Flow cytometry gating strategy. The total lymphocytes were gated on the forward/side scatter plot. Next, dead cells were excluded, then CD45^+^CD3^+^ CD4^+^ populations were selected, and subsequently plotted in a new graph to calculate the percentages of the Th1cells, Th17cells and Treg cells subsets.


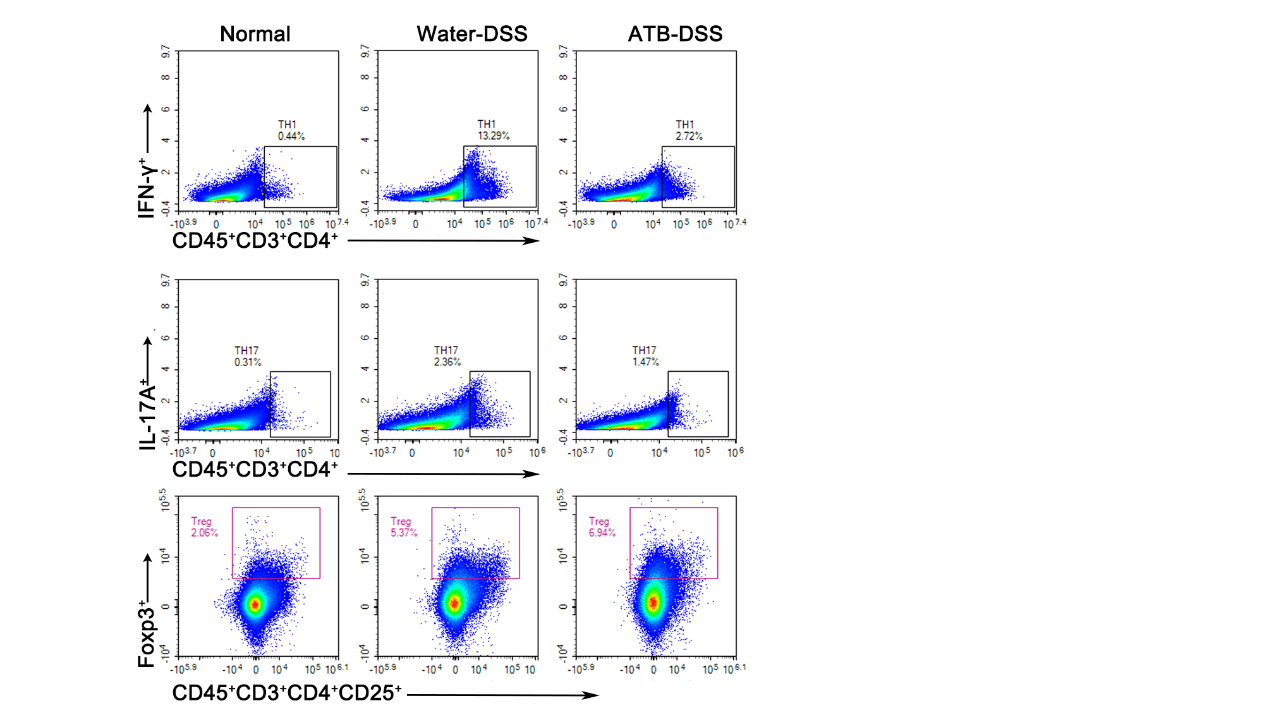


Figure S14. The results from the flow assay are presented. Mice were treated with antibiotics for 7 days, followed by induction of colitis using DSS. At the experimental endpoint, mesenteric lymph node cells were isolated and analyzed. The proportion of Th1 and Th17 cells in the antibiotic-treated (ATB-DSS) group was reduced compared to the DSS group, with a smaller reduction observed in Treg cells.


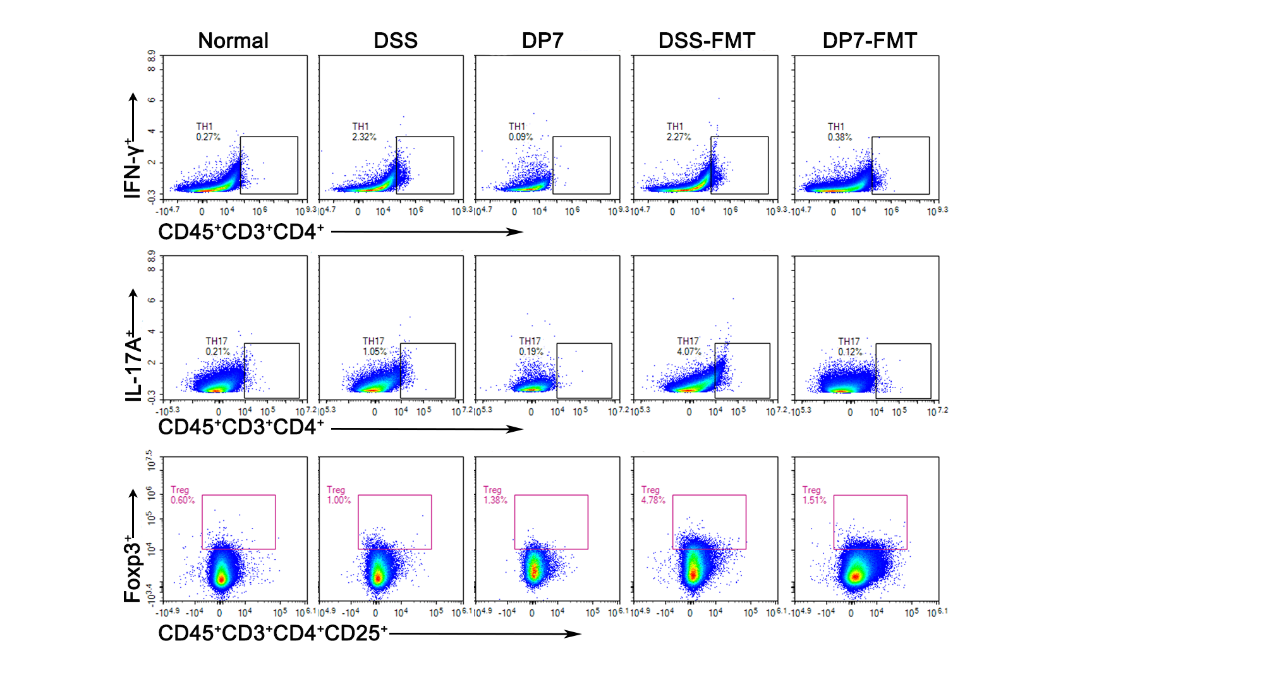


Figure S15. The results from the flow assay are presented. At the end of the experiment, mesenteric lymph node cells were isolated and analyzed. The proportions of Th1 and Th17 cells were reduced in the DP7 treatment group and the DP7-FMT treatment group compared to the DSS group, while the reduction in Treg cells was less pronounced.


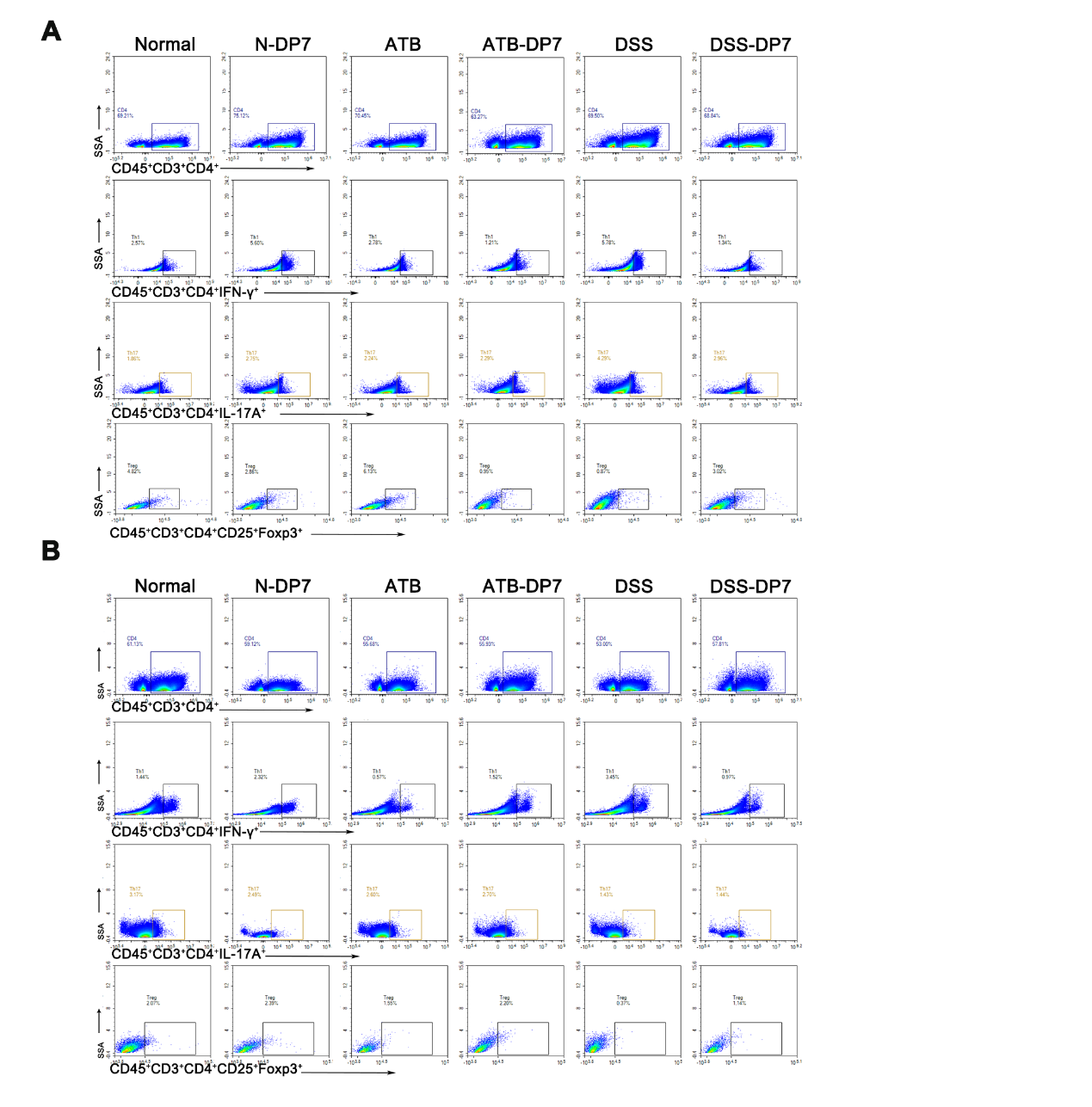


Figure S16. The results obtained from the flow cytometry assay are detailed and showed. DP7 was administered to normal mice, pseudo-germ-free mice, and colitis mice, and their splenic lymphocytes and mesenteric lymph node cells were subsequently examined. This study aimed to explore the effects of DP7 on the immune response and its relationship with gut microbiota.

Table 1

| **Target gene** | **Primer** | **Primer sequence (5′–3′)** |
| --- | --- | --- |
| β-actin | Forward | CCCAGGCATTGCTGACAGG |
| β-actin | Reverse | TGGAAGGTGGACAGTGAGGC |
| IL-1β | Forward | ATCTCGCAGCAGCACATCAA |
| IL-1β | Reverse | ACGGGAAAGACACAGGTAGC |
| IL-6 | Forward | CCAGTTGCCTTCTTGGGACT |
| IL-6 | Reverse | GTCTCCTCTCCGGACTTGTG |
| IL-10 | Forward | CATCGATTTCTTCCCTGTGAA |
| IL-10 | Reverse | TCTTGGAGCTTATTAAAGGCATTC |
| ZO-1 | Forward | GAGCCCCCTAGTGATGTGTG |
| ZO-1 | Reverse | TAGGGTCACAGTGTGGCAAG |
| occludin | Forward | TAGTGGCTTTGGCTACGGAGGT |
| occludin | Reverse | AGGAAGCCTTTGGCTGCTCTTG |
| claudin-1 | Forward | CCCTTCAGCAGAGCAAGGTT |
| claudin-1 | Reverse | TAGGGCAACCAAGTGCCTTT |
| Cxcl1 | Forward | CTGGGATTCACCTCAAGAACATC |
| Cxcl1 | Reverse | CAGGGTCAAGGCAAGCCTC |
| Cxcl2 | Forward | CCAACCACCAGGCTACAGG |
| Cxcl2 | Reverse | GCGTCACACTCAAGCTCTG |
| Ccl3 | Forward | TTCTCTGTACCATGACACTCTGC |
| Ccl3 | Reverse | CGTGGAATCTTCCGGCTGTAG |
| Ccl9 | Forward | CCCTCTCCTTCCTCATTCTTACA |
| Ccl9 | Reverse | AGTCTTGAAAGCCCATGTGAAA |
